# Early Archean origin of Photosystem II

**DOI:** 10.1111/gbi.12322

**Published:** 2018-11-09

**Authors:** Tanai Cardona, Patricia Sánchez‐Baracaldo, A. William Rutherford, Anthony W. Larkum

**Affiliations:** ^1^ Department of Life Sciences Imperial College London London UK; ^2^ School of Geographical Sciences University of Bristol Bristol UK; ^3^ University of Technology Sydney Ultimo New South Wales Australia

**Keywords:** Archean, Chloroflexi, Cyanobacteria, evolution, photosystem, Proteobacteria, reaction center, water oxidation

## Abstract

Photosystem II is a photochemical reaction center that catalyzes the light‐driven oxidation of water to molecular oxygen. Water oxidation is the distinctive photochemical reaction that permitted the evolution of oxygenic photosynthesis and the eventual rise of eukaryotes. At what point during the history of life an ancestral photosystem evolved the capacity to oxidize water still remains unknown. Here, we study the evolution of the core reaction center proteins of Photosystem II using sequence and structural comparisons in combination with Bayesian relaxed molecular clocks. Our results indicate that a homodimeric photosystem with sufficient oxidizing power to split water had already appeared in the early Archean about a billion years before the most recent common ancestor of all described Cyanobacteria capable of oxygenic photosynthesis, and well before the diversification of some of the known groups of anoxygenic photosynthetic bacteria. Based on a structural and functional rationale, we hypothesize that this early Archean photosystem was capable of water oxidation to oxygen and had already evolved protection mechanisms against the formation of reactive oxygen species. This would place primordial forms of oxygenic photosynthesis at a very early stage in the evolutionary history of life.

## 1 INTRODUCTION

The transition from anoxygenic to oxygenic photosynthesis initiated when an ancestral photochemical reaction center evolved the capacity to oxidize water to oxygen (Rutherford, 1989). Today, water oxidation is catalyzed in the Mn_4_CaO_5_ oxygen‐evolving cluster of Photosystem II (PSII) of Cyanobacteria and photosynthetic eukaryotes. How and when Type II reaction centers diversified, and how and when one of these reaction centers evolved the capacity to oxidize water are questions that still remain to be answered. While there is agreement that by 3.5 Ga (billion years before the present) a form of anoxygenic photoautotrophy had already evolved (Butterfield, 2015; Nisbet & Fowler, 2014; Tice & Lowe, 2004), the sedimentological and isotopic evidence for the origin of oxygenic photosynthesis has been interpreted to range from 3.7 Ga (Frei et al., 2016; Rosing & Frei, 2004) to the Great Oxidation Event (GOE) at ~2.4 Ga (Johnson et al., 2013). Molecular clock studies have generated a wider range of age estimates for the origin of Cyanobacteria spanning between 3.5 Ga (Falcon, Magallon, & Castillo, 2010) and <2.0 Ga (Betts et al., 2018; Shih, Hemp, Ward, Matzke, & Fischer, 2017). There is thus great uncertainty and no consensus. For this reason, determining when PSII evolved the capacity to oxidize water should greatly advance our understanding of the origin of oxygenic photosynthesis.

**Figure 1:**
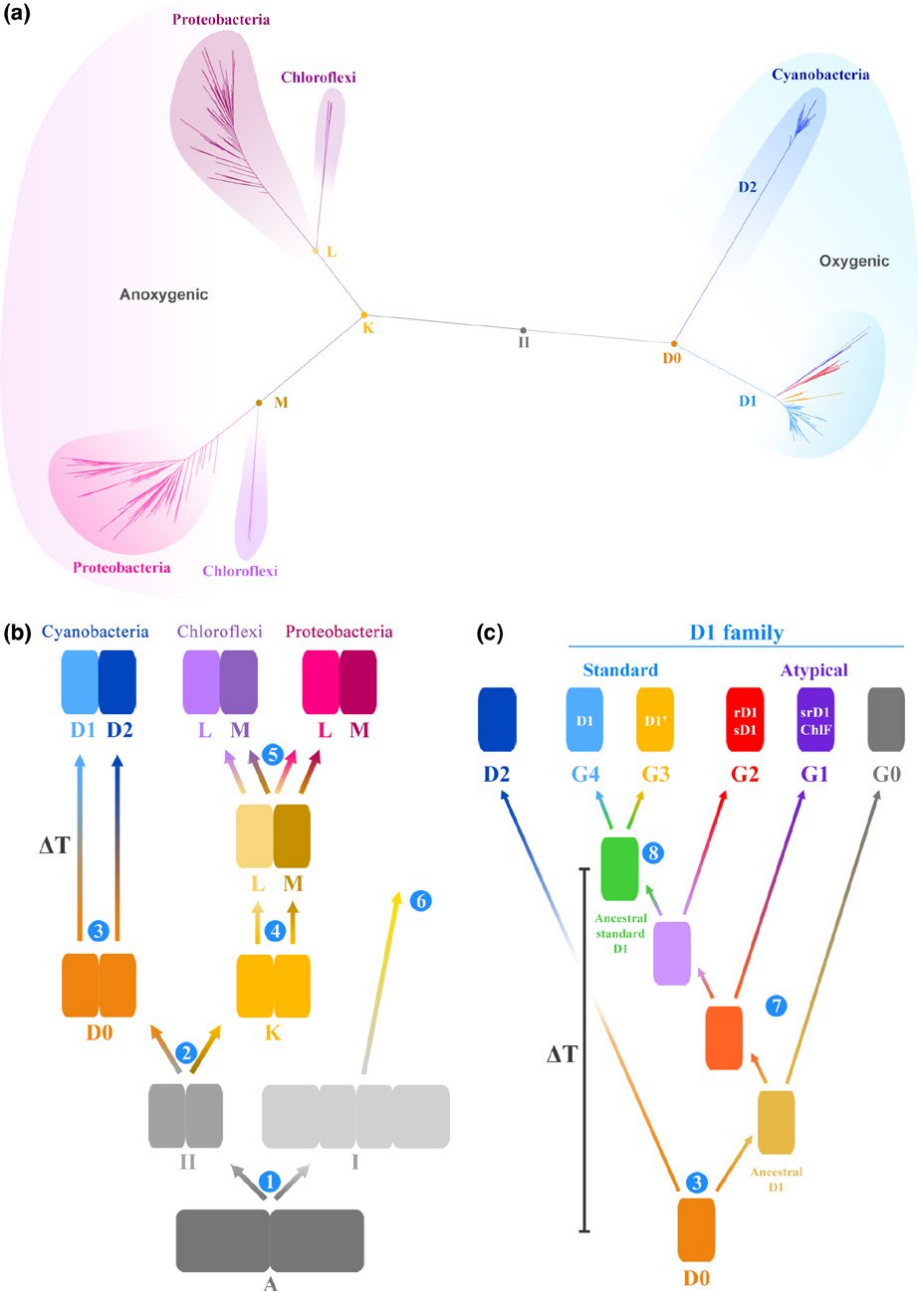
Evolution of Type II reaction center proteins. (a) A maximum likelihood phylogeny of Type II reaction center proteins. (b) A schematic representation of the phylogeny shown in (a). All reaction centers have common ancestry and descended from a homodimeric reaction center, marked A. From A, two new reaction centers emerged, one ancestral to all Type II reaction centers (II) and a second ancestral to all Type I reaction centers. This is the earliest diversification event of reaction center proteins that can be inferred from sequence and structural data and it is marked 1. The ancestral Type II reaction center protein (II) gave rise to two new proteins, one ancestral to D1 and D2, named here D0 and a second ancestral to L and M named K. The ancestral L and M subunits further diversify into Chloroflexi‐type and Proteobacteria‐type L and M subunits (5). Step 6 indicates that Type I reaction center proteins also diversified in parallel to Type II reaction center proteins. (c) Evolution of cyanobacterial D1 and D2, modified from Cardona et al. (2015). G0, G1, and G2 represent atypical D1 forms, and G3 and G4 standard D1 forms. ΔT marks the span of time between D0 and the appearance of the ancestral standard form of D1, which characterizes PSII and predates the most recent common ancestor of all known Cyanobacteria capable of oxygenic photosynthesis [Colour figure can be viewed at wileyonlinelibrary.com]

The evolution of Type II reaction center proteins has been described and discussed in some detail before (Beanland, 1990; Blankenship, 1992; Cardona, 2015, 2016; Nitschke & Rutherford, 1991; Rutherford & Nitschke, 1996; Sadekar, Raymond, & Blankenship, 2006) and it is presented and schematized in Figure 1. Type II reaction centers can be divided into two major families: the *oxygenic* and the *anoxygenic* Type II reaction centers. The oxygenic Type II reaction center is also known as PSII, and its electron transfer core is made of two homologous reaction center proteins, D1 and D2, exclusively found in Cyanobacteria and photosynthetic eukaryotes. The Mn_4_CaO_5_ cluster is bound by D1 and the core antenna protein CP43 (Ferreira, Iverson, Maghlaoui, Barber, & Iwata, 2004). On the other hand, anoxygenic Type II reaction centers are found in phototrophic members of the phyla Proteobacteria, Chloroflexi, and Gemmatimonadetes, with the latter obtaining the reaction center via horizontal gene transfer (HGT) from a gammaproteobacterium (Zeng, Feng, Medova, Dean, & Koblizek, 2014). The core subunits of the anoxygenic Type II reaction centers are known as L and M and lack an oxygen‐evolving cluster.

There is no doubt that D1, D2, L, and M share a common origin: Beanland (1990) was the first to record this but has been followed by many others (Cardona, 2015; Nitschke & Rutherford, 1991; Sadekar et al., 2006). That is to say that D1, D2, L and M, all descended from a single protein (denoted II in Figure 1). The earliest event in the evolution of Type II reaction centers can be described as the divergence of this ancestral protein into two new forms, one ancestral to D1 and D2, the *oxygenic* branch; and a second one ancestral to L and M, the *anoxygenic* branch (Figure 1). Hence, D1 and D2 originated from a gene duplication event and together make a monophyletic clade of Type II reaction center proteins, distinct from that which gave rise to L and M (Cardona, 2015, 2016). The ancestral protein to D1 and D2 will be referred to as D0 and the ancestral protein to L and M will be referred to as K.

As a result of the monophyletic relationship of D1 and D2 and the conserved structural and functional characteristics between these two proteins, it is possible to reconstruct traits of the ancestral photosystem. Some of the conserved traits, present in both D1 and D2, but absent in L and M, suggest that the ancestral homodimeric photosystem, made of a D0 dimer, was already unlike any of the known anoxygenic Type II reaction centers and had acquired characteristics associated with the highly oxidizing potential required for water oxidation (Cardona, 2016; Cardona, Sedoud, Cox, & Rutherford, 2012; Rutherford & Faller, 2003; Rutherford & Nitschke, 1996). One of these conserved traits is a redox tyrosine–histidine pair strictly conserved in both D1 and D2, Y_Z_‐H190 and Y_D_‐H189, respectively. The presence of these tyrosine–histidine pairs indicates that the midpoint potential (E_*m*_) of the photochemical chlorophylls at the heart of the reaction center was oxidizing enough to generate the neutral tyrosyl radical on either side of the homodimeric reaction center (Rutherford & Faller, 2003; Rutherford & Nitschke, 1996). That is an E_*m*_ of at least 1 V (DeFelippis, Murthy, Faraggi, & Klapper, 1989; DeFelippis et al., 1991), sufficient to drive the oxidation of water to oxygen, which has an E_*m*_ of 0.82 V at pH 7 (Dau & Zaharieva, 2009; Tachibana, Vayssieres, & Durrant, 2012). Based on this and other arguments, Rutherford and Nitschke (1996) suggested that before the gene duplication that led to D1 and D2, this ancestral photosystem was well on its way toward the evolution of water oxidation, and may have been able to oxidize water, even if only inefficiently.

Several types of D1 can be distinguished phylogenetically (Cardona, Murray, & Rutherford, 2015) and their evolution is schematized in Figure 1c. The early evolving forms, referred to as atypical D1 forms (G0, G1, G2 in Figure 1), are characterized by the absence of some, but not all, of the ligands to the Mn_4_CaO_5_ cluster and have been recently found to be involved in the synthesis of chlorophyll *f*, which supports oxygenic photosynthesis using low energy far‐red light (Ho, Shen, Canniffe, Zhao, & Bryant, 2016; Nurnberg et al., 2018); or the inactivation of PSII when anaerobic processes are being carried out (Murray, 2012; Wegener, Nagarajan, & Pakrasi, 2015). The late evolving forms, referred to as the standard D1 forms, are characterized by a complete set of ligands to the Mn_4_CaO_5_ cluster and are the main D1 used for water oxidation. Among the standard forms, there are also several types, which have been roughly subdivided into two groups: the microaerobic forms of D1 (G3) and the dominant form of D1 (G4). The microaerobic forms are suspected to be expressed only under low‐oxygen conditions. The dominant form, G4, is the main D1 used for water oxidation by all Cyanobacteria and photosynthetic eukaryotes. Most Cyanobacteria carry in their genomes an array of different D1 types, yet every strain has at least one dominant form of D1 (G4). Therefore, all Cyanobacteria descended from a common ancestor that already had evolved efficient oxygenic photosynthesis, had a dominant form of D1, and was able to assemble a standard PSII virtually indistinguishable from that of later evolving strains. Furthermore, because the atypical D1 forms support or regulate oxygenic photosynthesis under specific environmental conditions it can be argued that when these branched out water oxidation to oxygen had already evolved.

Based on the phylogeny of reaction center proteins, several stages in the evolution of oxygenic photosynthesis can be envisaged: The earliest of these stages is the divergence of Type I and Type II reaction center proteins (1, Figure 1b); this is then followed by the divergence of the *anoxygenic* family (L/M) and the *oxygenic* family (D1/D2) of Type II reaction center proteins (2), then by the duplication event that led to the divergence of D1 and D2 (3), and the subsequent (7) gene duplication events and specializations that created the known diversity of D1 forms, which ultimately resulted in the emergence of the standard form of D1. Because a photosystem made of a D0 had already acquired some of the fundamental features required to oxidize water such as highly oxidizing chlorophyll cofactors and the capacity to generate the neutral tyrosyl radical at each side of the reaction center: Then, it can be suggested that some of the earliest stages specific to the evolution of PSII and oxygenic photosynthesis had occurred between stages 2 and 3 as depicted in Figure 1b. Therefore, the span of time between D0 and the ancestral standard form of D1 (marked 8 in Figure 1c) represents the duration of the evolutionary trajectory of PSII from a simpler homodimeric highly oxidizing reaction center to the more complex enzyme inherited by all organisms capable of oxygenic photosynthesis. We denote this span of time by ΔT. If ΔT is small, such as a few million years or less for example, then the evolution of oxygenic photosynthesis may be better described as a sudden and fast process only getting started shortly before the GOE as suggested by some recent analyses (Shih, Hemp et al., 2017; Ward, Kirschvink, & Fischer, 2016). On the other hand, if ΔT is large: Several hundred million years or more for example, then the earliest stages in the evolution of oxygenic photosynthesis could significantly predate the GOE as suggested by some geochemical (Mukhopadhyay et al., 2014; Planavsky et al., 2014; Satkoski, Beukes, Li, Beard, & Johnson, 2015) and phylogenetic data (Blank & Sanchez‐Baracaldo, 2010; Schirrmeister, de Vos, Antonelli, & Bagheri, 2013).

Here, we report an in‐depth evolutionary analysis of Type II reaction center proteins including Bayesian relaxed molecular clocks under various scenarios for the origin of photosynthesis. The data presented here indicate that a photosystem with the structural and functional requirements to support the oxidation of water to oxygen could have arisen in the early Archean and long before the most recent common ancestor of Cyanobacteria.

## 2 RESULTS

### 2.1 Change in sequence identity as a function of time

**Figure 2:**
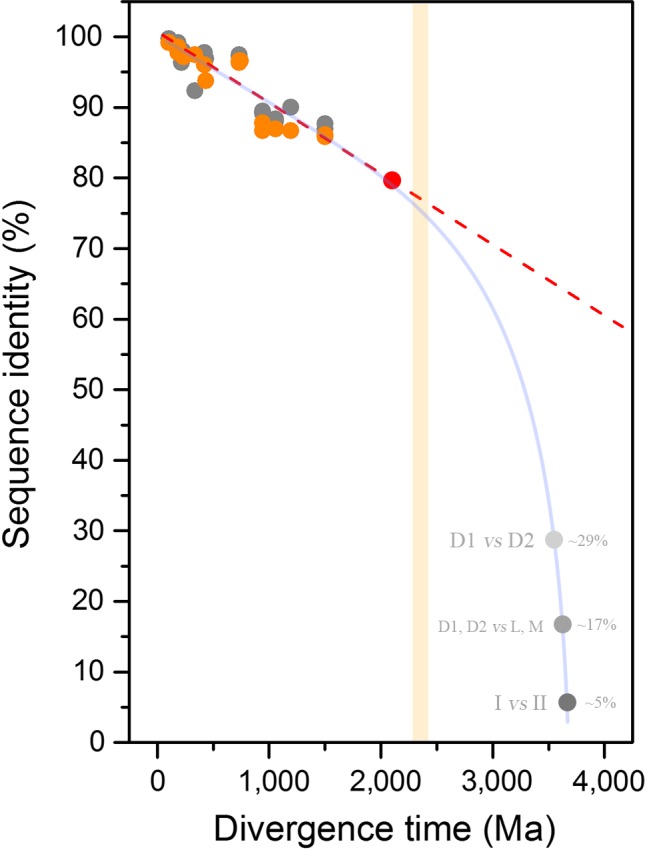
Decrease of sequence identity of D1 and D2 proteins as a function of divergence time. D1 subunits are shown in gray and D2 in orange. The divergence time between pairs of species is plotted against the level of sequence identity as tabulated in Supplementary Table S1. The red circle, placed at 79.2%, corresponds to the average sequence identity of the three distinct Group 4 D1 sequences of *Gloeobacter violaceous* in comparison with that of *Cyanidioschyzon merolae*. The light orange bar marks the GOE. The dashed line is fitted from a linear function and shows that over a period of at least 2.0 Ga, no dramatic changes in the rates of evolution of D1 and D2 are observed. The red dashed lines show an extrapolation of current rates of evolution throughout Earth's history. This line highlights that the rate is too slow for the divergence of D1 and D2 to have started right before the GOE. The gray dots around 3.5–3.8 Ga mark a speculative timing for the earliest events in the history of photosynthesis: the divergence of D1 and D2 (~29% sequence identity), the divergence of anoxygenic (L/M) and oxygenic (D1/D2) reaction center proteins (~17%), and the divergence of Type I and Type II reaction center proteins (≤10%). The curved blue line highlights that any scenario for the diversification of reaction centers after the origin of life requires faster rates of evolution at the earliest stages in the evolution of photosynthesis [Colour figure can be viewed at wileyonlinelibrary.com]

A first approximation to the evolution of Type II reaction centers as a function of time can be derived from the level of sequence identity between D1 and D2 of different species with known or approximated divergence times as shown in Figure 2. For example, the D1 protein of the dicotyledon *Arabidopsis thaliana* shares 99.7% amino acid sequence identity with that of the dicotyledon *Populus trichocarpa*, and these are estimated to have diverged between 127.2 and 82.8 Ma (Clarke, Warnock, & Donoghue, 2011), see Figure 2 and Supporting information Table S1. On the other hand, *A. thaliana*'s D1 shares 87.7% sequence identity with that of a unicellular red alga *Cyanidioschyzon merolae*. Complex multicellular red algae are known to have diverged at least 1.0 Ga ago (Butterfield, 2000; Gibson et al., 2017) and recently described fossils could push this date back to 1.6 Ga (Bengtson, Sallstedt, Belivanova, & Whitehouse, 2017; Sallstedt, Bengtson, Broman, Crill, & Canfield, 2018). At the other end of this evolutionary line, the three dominant forms of D1 from *Gloeobacter violaceous* (G4) share on average 79.2% sequence identity with that of *C. merolae* or 78.5% with that of *A. thaliana*. If the percentage of sequence identity between pairs of species is plotted as a function of their divergence time, a linear decrease of identity is observed among reaction center proteins at a rate of less than 1% per 100 million years (Supporting information Table S2). The trend in Figure 2 indicates that the rate of evolution of D1 and D2 since the GOE and since the emergence of photosynthetic eukaryotes has remained very slow and stable until the present time, if considered over a large geological time scale, with less than 20% change in sequence identity in the past 2.0 Ga.

Now, if the most recent common ancestor (MRCA) of Cyanobacteria capable of oxygenic photosynthesis, defined as the MRCA of the genus *Gloeobacter* and all other extant photosynthetic strains, existed hundreds of millions of years before the GOE, this would presuppose an even slower rate of evolution of the core subunits of PSII. In contrast, if the rate of evolution of D1 and D2 are taken at face value, following the roughly uniform rate observed in photosynthetic eukaryotes, this would locate the divergence of *Gloeobacter* after the GOE (Figure 2, red spot): In consequence, the older the MRCA of Cyanobacteria, the slower the rate of evolution of the dominant form of D1 and D2. Therefore, large uncertainties in the fossil record of photosynthetic eukaryotes would result in only small changes to this trend. For example, if the divergence of red algae occurred as late as 1.0 Ga or as early as 2.0 Ga, this will only cause a small shift in the overall rate. Or for example, if the MRCA of angiosperms is actually 100 million years older than currently understood, this would result in almost a negligible change in the rate of evolution of the dominant form of D1 and D2 compared over the large time scale of the planet.

Let us reiterate that all the evidence suggests that all reaction center proteins originated from a single ancestral protein that diversified as the multiple groups of photosynthetic bacteria arose. As a result of this common ancestry, any standard D1 shares on average about 29% sequence identity with any D2 across their entire sequence. Any standard D1 or D2 shares on average 17% sequence identity with any L or M. The level of sequence identity falls well below 10% if any Type II reaction center protein is compared with any Type I reaction center protein (Cardona, 2015). As a result of this, the rate of evolution of D1 and D2 since the GOE, as estimated from the decrease of sequence identity (<1% per 0.1 Ga), is too slow to account for the evolution of photochemical reaction centers within a reasonable amount of time (Figure 2, dashed line). In other words, the rate of evolution of reaction center proteins since the origin of life could not have been constant, and any scenario for the origin of photochemical reaction centers at any point in the Archean requires initially faster rates of evolution than any rate observed since the Proterozoic (Figure 2, light blue line).

Taking into consideration that D1 and D2 share only about 29% sequence identity, two other observations can be made, as illustrated in Figure 2: (a) that the duplication that led to the divergence of D1 and D2 is more likely to have occurred closer to the origin of the primordial reaction center proteins at the origin of photosynthesis in the early Archean, than closer to or after the GOE; and (b) that the MRCA of Cyanobacteria is more likely to have existed closer to the GOE than closer to the origin of photosynthesis.

### 2.2 Bayesian relaxed molecular clock analysis

**Figure 3:**
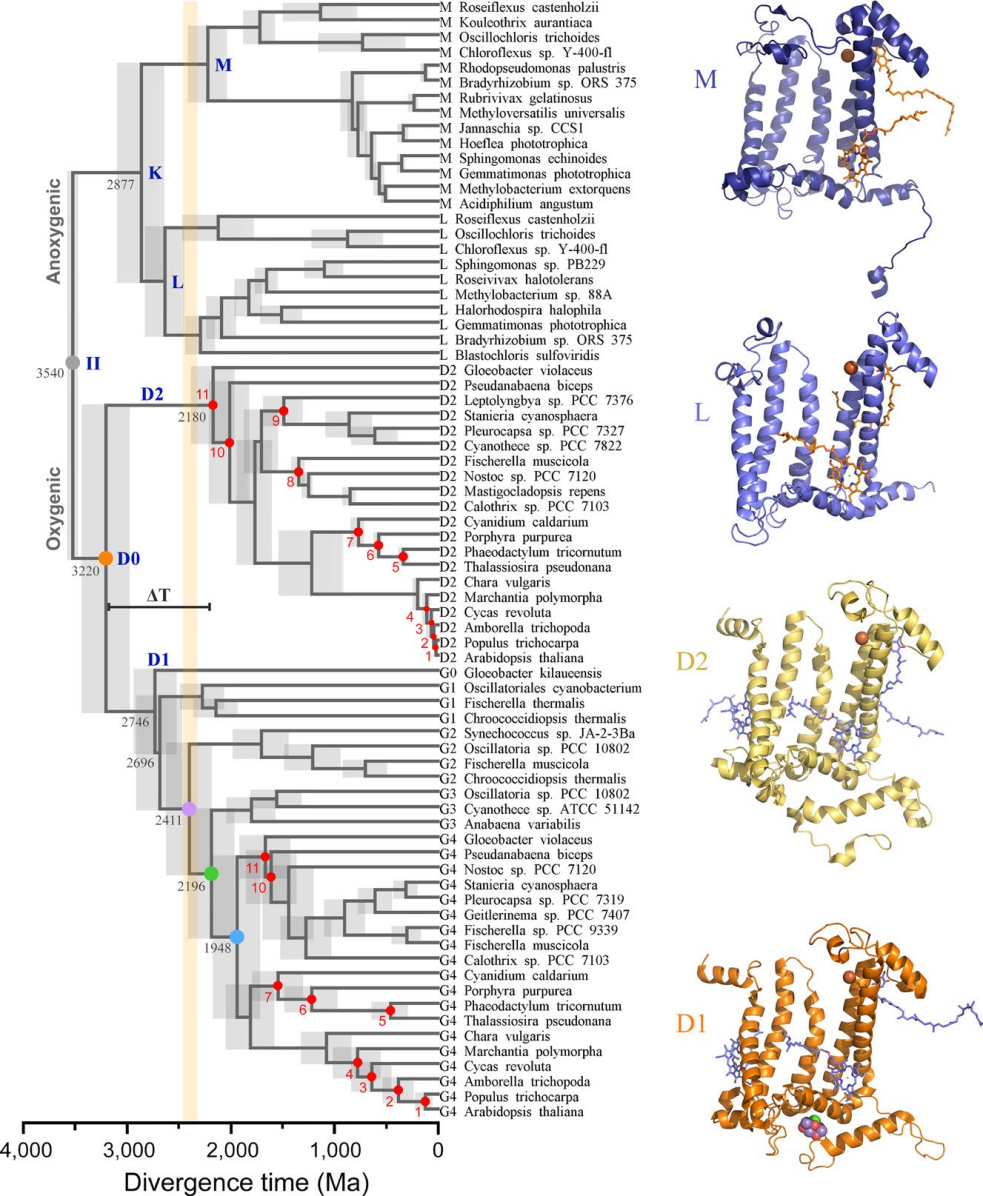
Relaxed molecular clock of Type II reaction center proteins. A log‐normal autocorrelated relaxed clock is shown implementing a CAT + GTR + Γ model with flexible boundaries on the calibration points. Red dots are calibration points as described in Materials and Methods. The gray dot denoted II represents the ancestral Type II reaction center protein, as schematized in Figure 1. The orange dot (D0) marks the initial divergence of D1 and D2. The violet dot marks the divergence point between G2 atypical D1 sequences and standard D1. The green dot marks the divergence point between the microaerobic D1 forms (G3) and the dominant form of D1 (G4). This point represents the last common ancestral protein to all standard D1 forms predating crown group Cyanobacteria. The blue dot represents the origin of the dominant form of D1 inherited by all extant Cyanobacteria and photosynthetic eukaryotes. The gray bars represent the standard error of the estimated divergence times at the nodes. The orange bar shows the GOE [Colour figure can be viewed at wileyonlinelibrary.com]

**Figure 4:**
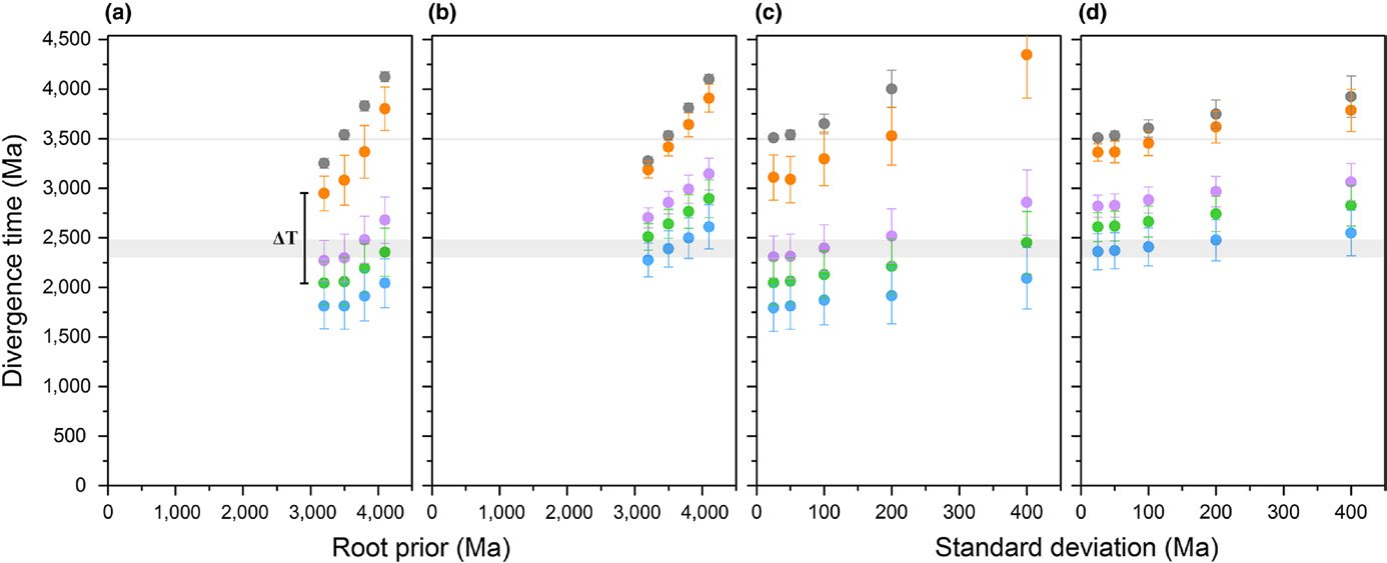
Effect of model selection on estimated divergence times. (a) Divergence times of key nodes in the evolution of Type II reaction centers as a function of the root prior. The root prior was varied from 3.2 to 4.1 ± 0.05 Ga under a CAT + GTR + Γ model. The colored dots match selected nodes of interest in Figure 3. The thick gray bar marks the GOE and the narrow bar marks the minimum accepted age for the origin of photosynthesis. (b) Identical to (a) but using a LG + Γ model of amino acid substitutions. (c) Divergence times of key nodes assuming a root prior of 3.5 Ga as a function of the standard deviation on the root. The standard deviation was varied from 0.025 to 0.4 Ga under a CAT + GTR + Γ model. (d) Identical to (c) but using a LG + Γ model. In every case, D0 is the oldest node after the root and the magnitude of ΔT is always in the range of a billion years [Colour figure can be viewed at wileyonlinelibrary.com]

The simple approach used above indicates that the divergence of D1 and D2 is likely placed well before the GOE, to confirm this observation we applied a molecular clock to the phylogeny of Type II reaction center proteins. Figure 3 shows a Bayesian relaxed log‐normal autocorrelated molecular clock built using the CAT + GTR + Γ model allowing for flexible boundaries on the calibration points (Lartillot, Lepage, & Blanquart, 2009). As an informed starting point, we first specified the age of the root (root prior) at 3.5 Ga with a standard deviation of 0.05 Ga. That is to say, that the most ancestral form of a Type II reaction center protein is assumed to have already evolved by 3.5 Ga. Under these conditions, the last common ancestral protein to the standard form of D1 prior to the divergence of the G3 and G4 types (Figure 3, green dot) is timed at 2.19 ± 0.22 Ga. On the other hand, D0 (Figure 3, orange dot) is timed at 3.22 ± 0.19 Ga. It follows then that the difference in time between D0 and the first standard form of D1, ΔT, is 1.02 Ga, with the level of uncertainty on the estimated ages resulting in a range for ΔT between 1.44 and 0.60 Ga (see Table 1 and Figure 4a and b). This large ΔT agrees with the predictions made from the comparisons of sequence identity plotted in Figure 2.

**Table 1:** Effect on ΔT assuming different ages for the most ancestral Type II reaction center proteins

Root prior (Ga)	II	D0	Ancestral standard D1	ΔT (range)
CAT + GTR + Γ				
3.2	3.25 ± 0.05	2.80 ± 0.16	1.99 ± 0.19	0.80 (1.17–0.44)
3.5	3.54 ± 0.05	3.22 ± 0.19	2.19 ± 0.24	1.02 (1.44–0.60)
3.8	3.83 ± 0.05	3.44 ± 0.21	2.27 ± 0.24	1.17 (1.62–0.71)
4.1	4.12 ± 0.05	3.71 ± 0.23	2.38 ± 0.25	1.32 (1.81–0.84)
CAT + GTR + Γ and removing calibration point 11				
3.5	3.52 ± 0.05	3.00 ± 0.29	1.78 ± 0.25	1.22 (1.77–0.66)
3.8	3.81 ± 0.05	3.15 ± 0.29	1.77 ± 0.25	1.37 (1.93–0.81)
LG + Γ				
3.2	3.27 ± 0.05	3.19 ± 0.08	2.51 ± 0.13	0.68 (0.89–0.46)
3.5	3.53 ± 0.05	3.40 ± 0.09	2.64 ± 0.15	0.77 (1.00–0.53)
3.8	3.81 ± 0.05	3.64 ± 0.12	2.77 ± 0.17	0.88 (1.16–0.58)
4.1	4.10 ± 0.05	3.91 ± 0.14	2.90 ± 0.19	1.01 (1.34–0.68)
LG + Γ and removing calibration point 11				
3.5	3.49 ± 0.05	3.18 ± 0.19	2.30 ± 0.20	0.87 (1.26–0.48)
3.8	3.79 ± 0.05	3.52 ± 0.19	2.55 ± 0.22	1.38 (0.97–0.55)

To test the effect of different root priors on our results, we varied the age of the root and the standard deviation over a broad range. Table 1 lists estimates of divergence times of key ancestral Type II reaction center proteins and the respective ΔT value using different root priors. For example, under the assumption that Type II reaction centers had already evolved by 3.8 Ga ago (Czaja et al., 2013; Nisbet & Fowler, 2014; Rosing, 1999), ΔT is found to be centered at 1.17 Ga. Similarly, if it is assumed to be a late event occurring at 3.2 Ga, though unlikely, ΔT is still 0.80 Ga. Furthermore, increasing the standard deviation on the root prior pushes the timing of the earliest events in the evolution of Type II reaction centers to even older ages rather than younger ages, see Table 2 and Figure 4c and d. For example, a root prior of 3.5 Ga with a standard deviation of 0.1 Ga pushes the estimated time for the root to 3.65 Ga, making D0 3.30 ± 0.27 and generating a ΔT of over a billion years.

**Table 2:** Effect of varying the standard deviation (*SD*) on the root prior at 3.5 Ga

*SD* (Ga)	II	D0	Ancestral standard D1	ΔT (range)
CAT + GTR + Γ				
0.025	3.50 ± 0.03	2.99 ± 0.22	2.07 ± 0.22	0.91 (1.31–0.46)
0.05	3.54 ± 0.05	3.22 ± 0.19	2.19 ± 0.24	1.02 (1.44–0.60)
0.10	3.65 ± 0.10	3.29 ± 0.26	2.22 ± 0.23	1.07 (1.56–0.57)
0.20	4.00 ± 0.19	3.61 ± 0.32	2.32 ± 0.25	1.32 (1.87–0.70)
0.40	4.82 ± 0.38	4.55 ± 0.44	2.50 ± 0.28	1.74 (2.48 – 1.01)
LG + Γ				
0.025	3.51 ± 0.02	3.36 ± 0.09	2.61 ± 0.15	0.75 (0.98–0.51)
0.05	3.53 ± 0.05	3.37 ± 0.11	2.62 ± 0.15	0.75 (1.00–0.48)
0.10	3.60 ± 0.09	3.45 ± 0.13	2.67 ± 0.16	0.79 (1.07–0.50)
0.20	3.75 ± 0.14	3.62 ± 0.16	2.74 ± 0.18	0.88 (1.21–0.53)
0.40	3.92 ± 0.21	3.79 ± 0.21	2.83 ± 0.21	0.96 (1.37–0.53)

The Bayesian clock using flexible boundaries on the calibration points consistently produced ages for the divergence of the D2 subunit of *G. violaceous* and the dominant form of D1 (G4) after the GOE, similar to the ages reported by Shih, Hemp et al. (2017). Yet, previous molecular clocks have suggested that the MRCA of Cyanobacteria might predate the GOE (Schirrmeister, Gugger, & Donoghue, 2015), so we also performed a similar analysis that allowed us to explore this scenario. This was achieved using an empirical amino acid substitution model (LG + Γ) instead of the non‐parametric approach described above. We found this to be the only way to locate the D2 of *G. violaceous* and the dominant form of D1 (G4) before the GOE. The effect of less flexible boundaries on the estimated divergence times is shown in Tables 1 and 2, and Figure 4b and d. For example, assuming a root at 3.5 ± 0.05 Ga, the estimated divergence time for the standard form of D1 becomes 2.64 ± 0.15 Ga and pushes D0 back to 3.40 ± 0.09 Ga, making ΔT 0.77 Ga. On the other hand, if we allowed flexibility on the root prior by increasing the standard deviation to 0.4 Ga (Table 2), the estimated divergence time for the standard form of D1 becomes 2.83 ± 0.21, but the estimated age of the root is pushed back to 3.92 ± 0.21 Ga with D0 at 3.79 ± 0.21, making ΔT about a billion years. Overall, placing the MRCA of Cyanobacteria before the GOE pushes the gene duplication event that led to the divergence of D1 and D2 even closer to the origin of Type II reaction centers and to the origin of photosynthesis, just as predicted by the comparison of the level of sequence identity.

### 2.3 Rates of evolution

**Figure 5:**
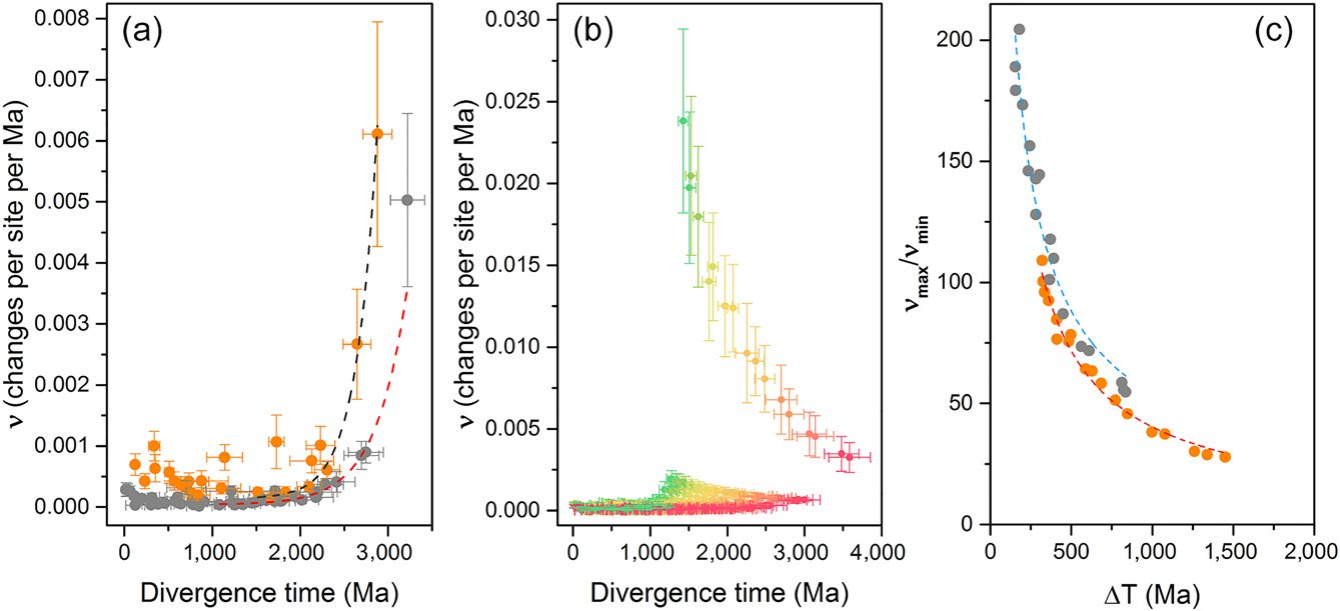
Rates of evolution as a function of time. (a) Change in the rate of evolution of oxygenic (gray) and anoxygenic (orange) Type II reaction center proteins. The rates correspond to the tree in Figure 3, assuming an origin of photosynthesis at about 3.5 Ga. The dashed lines represent a fit of a single‐component exponential decay and the rates are given as amino acid substitutions per site per million years. (b) Changes in the rate of evolution constraining the root to younger and younger ages. The red curve farthest to the right was calculated using a root prior of 4.2 ± 0.05 Ga, while the green curve farthest to the left was calculated using a root prior of 0.8 ± 0.05 Ga. Younger divergence times imply initial faster rates of evolution. (c) Change in the rate of evolution as a function of ΔT, the dashed lines represent a fit to a power law function. The curve in orange was calculated using ΔT values subtracting the mean average of the divergence times of D0 and the ancestral standard D1. The curve in gray was calculated using ΔT values subtracting the minimum age of D0 and the maximum age for ancestral standard D1 [Colour figure can be viewed at wileyonlinelibrary.com]

The inferences derived from Figure 2 revealed that the rates of evolution had to be faster in the initial stages during the Archean compared with the Proterozoic, even when ΔT is as large as one billion years. To gain a better understanding of the changes of the rate of evolution of Type II reaction center proteins, we plotted the rates as a function of divergence time. In Figure 5a, the rate of evolution (*ν*) of each node in the tree, expressed as amino acid substitutions per site per unit of time, is plotted against the estimated divergence time for each respective node. It can be seen that the rate at the earliest stage is much faster than the rates observed since the Proterozoic. Thus, faster rates are necessary to explain the origin and evolution of Type II reaction centers at any point in the Archean and regardless of when exactly photosynthesis originated, as seen in Figure 5b. The decrease in the rate of evolution is consistent with the observations derived from Figure 2 and can be roughly fitted with a first‐order exponential decay curve (fitting parameters are presented in Supporting information Table S3). Figure 5a additionally shows that L and M have been evolving at a faster rate than D1 and D2. From this slow‐down of the rates, it can be calculated that since each respective duplication event (stages 2 and 4 in Figure 1) it took about 168 million years for D1 and D2 to fall to 50% sequence identity and about 115 million years for the same to occur for L and M.

The maximum rate of evolution in the D1 and D2 family of reaction center proteins is placed at the node that represents D0. We will refer to this rate as *ν*_max_. Figure 5a shows that the rate of evolution flattens out to comparatively slow rates during the Proterozoic. These rates correspond to the rates of Group 4 D1 and D2. We will refer to the average rate of evolution during this zone of slow change as *ν*_min_ and it is calculated as the average rate from each node in Group 4 D1 and D2. In Figure 5a, *ν*_max_ is 5.03 ± 1.42 amino acid substitutions per site per Ga, while *ν*_min_ is 0.12 ± 0.04 substitutions per site per Ga. Therefore, if Type II reaction centers had evolved by 3.5 Ga, to account for the divergence of D1 and D2 in one billion years, the initial rate of evolution had to be about 40 times larger than that observed since the MRCA of Cyanobacteria.

Table 3 lists the rates of evolution of a diverse number of proteins reported in independent studies in a broad range of organisms. It is found that the rate of evolution of the core subunits of PSII (*ν*_min_) is similar to the rate of other proteins that are billions of years old and highly conserved such as subunits of the ATP synthase, the cytochrome *b*_6_ *f* complex, or the ribosome. Our estimated rates fall well within the expected range of other cyanobacterial proteins, thus validating our calibration choices and consistent with the expected level of sequence identity as derived from Figure 2. Furthermore, even *ν*_max_ is found to be within plausible levels when ΔT is about a billion years: slower than known fast evolving proteins such as peptide toxins (Duda & Palumbi, 1999), the influenza virus (Carrat & Flahault, 2007), or proteins of the immune system (Hughes, 1997; Table 3).

**Table 3:** Comparison of rates of protein evolution

	Rate (Amino acid substitutions per site per Ga)	References
D0 (*ν*_max_)	5.03	This work^a^
Group 4 D1 and D2 (*ν*_min_)	0.12	This work^a^
K	6.11	This work^a^
L and M	0.61	This work^a^
PsaA, Photosystem I core subunit (Cyanobacteria)	0.09	Sanchez‐Baracaldo (2015)
AtpA, ATP Synthase CF1 Alpha Chain (Cyanobacteria)	0.08	Sanchez‐Baracaldo (2015)
PetB, Cytochrome *b*6 (Cyanobacteria)	0.05	Sanchez‐Baracaldo (2015)
RpsM, S13 Ribosomal Protein Translation (Cyanobacteria)	0.13	Sanchez‐Baracaldo (2015)
L1 Ribosomal Protein (Cyanobacteria)	0.11	Sanchez‐Baracaldo (2015)
ADP‐glucose pyrophosphorylase large subunit (plants)	1.2	Georgelis, Braun, and Hannah (2008)
PRTB Protein^b^ (humans)	0.13	Matsunami, Yoshioka, Minoura, Okano, and Muto (2011)
Alcohol dehydrogenase (ascidians)	0.27	Canestro et al. (2002)
Protein‐L‐isoaspartyl (D‐aspartyl) *O*‐methyltransferase (bacteria to humans^c^)	0.39	Kagan, McFadden, McFadden, O'Connor, and Clarke (1997)
Peptide neurotoxins (gastropods)	17	Duda and Palumbi (1999)
Hepatitis C Virus	3,700	Bukh et al. (2002)
Influenza virus type A (H1)	5,800	Carrat and Flahault (2007)

a Estimated using a root prior of 3.5 Ga under a autocorrelated log‐normal molecular clock as described in the text and Materials and Methods.

b Proline‐rich transcript overexpressed in the brain (PRTB). The human protein shares 99% sequence identity compared to that in mice. Rodents are estimated to have diverged about 74 Ma ago (Kay & Hoekstra, 2008).

c The authors pointed out that the rate of evolution of this methyltransferase has remained unchanged from bacteria to humans.

A rate of evolution of 0.12 substitutions per site per Ga, as seen for standard D1 and D2, means that it would take about 8 billion years for each position of the sequence to have changed at least once, assuming—just for the sake of simplicity—that each position has a similar chance of mutating. This very slow rate of evolution is the reason why standard D1 and D2 have changed little in more than 2.0 Ga, as seen in Figure 2. In contrast, the fast evolving peptide neurotoxins of the venomous gastropod *Conus* have been estimated to evolve at a rate of about 17 substitutions per site per Ga, which is about 140 times faster than D1 and D2. It means that each position in the sequence is expected to have changed at least once after only 60 Ma. In other words, it would take about 60 Ma for two identical neurotoxin peptides to lose all sequence identity. Unlike the slow‐evolving and highly conserved proteins of bioenergetics (including D1 and D2), which are under strong purifying selection, neurotoxins, viruses, or the immune system has evolved to generate change at the amino acid level within a few generations or within the lifetime of the organism. From these comparisons in the rates, it can be concluded that the homodimeric stage (D0) was likely very short‐lived, even when ΔT is in the order of a billion years.

Our Bayesian analyses show that the evolution of PSII is better described by a long span of time since the appearance of a homodimeric photosystem (with sufficient power to oxidize water) until the emergence of standard PSII (inherited by all known Cyanobacteria capable of photosynthesis). Notwithstanding, a fast rate of evolution at the earliest stage implies that *ν*_max_ would increase if ΔT is considered to be smaller, as would be the case for an evolutionary scenario in which PSII evolves rapidly before the GOE after an event of gene transfer of a bacteriochlorophyll *a*‐based anoxygenic Type II reaction center with low oxidizing power (i.e., before the evolution of tyrosine oxidation) like those found in phototrophic Proteobacteria and Chloroflexi (Shih, Hemp et al., 2017; Soo, Hemp, Parks, Fischer, & Hugenholtz, 2017). We illustrate this concept in Figure 5b and c. These figures depict the change of the rate of evolution as a function of ΔT. This manipulation of the molecular clock can only by accomplished computationally by changing the root prior to younger and younger ages. The increase of *ν*_max_ with decreasing ΔT can be fitted using a power law function (see Supporting information Table S4 for fitting parameters). This function can then be used to calculate *ν*_max_ under varying ΔT.

For example, Ward et al. (2016) calculated that the planet could have become oxygenated within just one hundred thousand years from the origin of oxygenic photosynthesis. Thus, in a hypothetical scenario in which a non‐photosynthetic ancestor of Cyanobacteria obtained photosynthesis via HGT from an anoxygenic phototroph, and this transferred reaction center evolved standard levels of oxygen evolution within one hundred thousand years (ΔT = 0.1 Ma), then *ν*_max_ would need to be more than 400 thousand amino acid changes per site per Ga, which is orders of magnitude greater than the rate of evolution of any known protein (Table 3). If ΔT is hypothesized to be 100 Ma instead, this would require a *ν*_max_ of about 33 amino acid substitutions per site per Ga, while less extreme, it is still twice the rate of short peptide neurotoxins. Unlike neurotoxins, photosynthetic reaction centers are highly regulated large multisubunit membrane protein complexes binding dozens of cofactors at precise orientations and distances to allow efficient photochemistry to occur. Even the “simplest” known homodimeric reaction center is made of at least four protein subunits binding 62 chlorophyll‐derived pigments, two carotenoids, two lipids, four Ca^2+^ ions, and a Fe_4_S_4_ cluster (Gisriel et al., 2017). It is therefore likely that reaction centers have always been under strong purifying selection (Shi, Bibby, Jiang, Irwin, & Falkowski, 2005). In fact, even the scenario in which ΔT is a billion years (*ν*_max_ = 5.03 ± 1.42) may be an overestimation and could potentially indicate that the age of the duplication event that led to D1 and D2 occurred immediately after the origin of the earliest reaction center. In consequence, the evolution of the core subunits of PSII is more consistent with a scenario in which oxygenic photosynthesis originated long before the GOE as supported by the geochemical record of inorganic redox proxies (Crowe et al., 2013; Havig, Hamilton, Bachan, & Kump, 2017; Planavsky et al., 2014; Wang et al., 2018).

### 2.4 The D1/D2 duplication is older than the L/M duplication

In Figure 3, it can also be seen that the divergence of the L and M subunits occurs *after* the divergence of D1 and D2. The estimated time for the divergence of L and M is 2.87 ± 0.16 Ga, while the time for the divergence of D1 and D2, as we saw above, is 3.22 ± 0.19 Ga. Because no calibration points were set on L and M, greater levels of uncertainty are observed in this part of the tree: Hence, we refrain from making strong inferences on the timing of specific diversification events within phototrophic Proteobacteria or Chloroflexi and only focus on the general trends. Still, we found the above result puzzling as it would place the roots of PSII before the roots of anoxygenic Type II reaction centers. After a closer inspection, we noted that this effect is caused by faster rates of evolution computed for L and M, relative to D1 and D2, across all time points (Figure 5a and Table 3). In consequence, at a faster rate of evolution, it would take less time for L and M to converge to node K than D1 and D2 to node D0. What is more, the phylogeny of Type II reaction centers, as seen in Figure 1, also shows that L and M branches are overall longer than D1 and D2 branches, which is suggestive of accelerated rates. Longer branches can be caused by a relatively early diversification due to a slow rate of evolution, or alternatively by a late diversification indicating comparatively faster rates. One question remains: Is this result an artifact of phylogenetic reconstruction given the lack of constraints on L and M, or does it have biological significance?

That anoxygenic phototrophs are displaying higher rates of evolution than Cyanobacteria is supported by other independent molecular clock studies (Magnabosco, Moore, Wolfe, & Fournier, 2018; Shih, Ward, & Fischer, 2017). For example, the level of sequence identity between L in *Roseiflexus castenholzii* and L in *Chloroflexus* sp. Y‐400‐fl, two relatively distant phototrophs of the phylum Chloroflexi, is about 45%. In comparison, the level of sequence identity between standard D1 in *Gloeobacter* and D1 in *Arabidopsis* is just under 80%, as we saw above. Shih, Ward et al. (2017) calculated that the MRCA of phototrophic Chloroflexi occurred about 1.0 Ga ago. That date would imply that the L in *Roseiflexus* and *Chloroflexus* lost 55% sequence identity since their most recent common ancestor about 1.0 Ga ago (1% loss for every ~18 Ma). If the estimated age reported by Shih, Ward et al. (2017) is correct, that would make the rate of evolution of L in the Chloroflexi about 5.5 times faster than D1 or D2 (1% loss for every ~100 Ma assuming *Gloeobacter* branched out 2.0 Ga ago). Magnabosco et al. (2018) computed an age for the MRCA of phototrophic Chloroflexi of 2.1 Ga (obtained with their Model D), which would make the rates of evolution of L 2.6 times faster than D1 and D2. The average rate of evolution for L and M calculated by our molecular clock is 0.61 ± 0.19 substitutions per site per Ga, while that for D1 and D2 is 0.12 ± 0.04 (see Table 3). Therefore, according to our clock L and M are evolving on average 4.7 times faster than D1 and D2. This result is nicely within the range suggested by the two independent studies referenced above and confirms that our approach using a single protein produced similar rates of evolution as those computed using a large set of highly conserved concatenated sequences (Magnabosco et al., 2018; Sanchez‐Baracaldo, 2015; Shih, Hemp et al., 2017).

It is worth noting here that under every scenario tested in this study, the duplication leading to D1 and D2 was always found to be the oldest node after the root. The late divergence of L and M relative to D1 and D2 does not seem to be artifactual but a consequence of the apparent faster rates of evolution measured in anoxygenic phototrophs. A ramification of this is that the hypothesis that Cyanobacteria obtained a Type II reaction center via HGT from an anoxygenic phototroph, right before the GOE, becomes untenable because D1 and D2 would predate L and M.

### 2.5 Sensitivity analysis

To test the reliability of the method, we explored a range of contrasting models. We compared the effect of the model of relative exchange rates on the estimated divergence times: Supporting information Figure S1 provides a comparison of estimated divergence times calculated with the CAT + GTR model (Yang & Rannala, 2006) against divergence times calculated using the CAT model with a uniform (Poisson) model of equilibrium frequencies. The GTR model does not have a strong effect on the calculated divergence times as the slope of the graph does not deviate from unity when paired with the uniform model (see Supporting information Table S5 for linear regressions). Thus under a root prior of 3.5 ± 0.05 Ga, a CAT + Poisson model also generated a ΔT centered at 1.02 Ga, see Table 4.

**Table 4:** Change in ΔT under different evolutionary models

Model	Root prior (Ga)	Calibration (Ga)	ΔT (Ga)
CAT + GTR (autoc.^a^)	3.5	2.45	1.02 (1.44–0.52)
CAT + GTR (autoc.)	3.5	2.70	0.97 (1.29–0.64)
CAT + GTR (autoc.)	3.8	2.45	1.19 (1.64–0.70)
CAT + GTR (autoc.)	3.8	2.70	1.13 (1.48–0.76)
CAT + Pois. (autoc.)	3.5	2.45	1.02 (1.51–0.52)
CAT + Pois. (autoc.)	3.5	2.70	1.00 (1.34–0.66)
CAT + Pois. (autoc.)	3.8	2.45	1.17 (1.68–0.70)
CAT + Pois. (autoc.)	3.8	2.70	1.12 (1.50–0.75)
CAT + Pois. (uncor.^b^)	3.5	2.45	1.94 (2.62 ‐1.26)
CAT + Pois. (uncor.)	3.5	2.70	1.84 (2.46–1.22)
CAT + Pois. (uncor.)	3.8	2.45	1.67 (2.31–1.03)
CAT + Pois. (uncor.)	3.8	2.70	2.03 (2.70–1.38)

a Log normal autocorrelated clock model.

b Uncorrelated gamma model.

To understand the effects of the oldest calibration point (point 11, Figure 3) on the estimated divergence time, we tested a second set of boundaries restricting this point to a minimum age of 2.7 Ga (Calibration 2) to consider the possibility that the record for oxygen several hundred million years before the GOE was produced by crown group Cyanobacteria (Havig et al., 2017; Planavsky et al., 2014). Supporting information Figure S2 provides a comparison of the two calibration choices on the overall estimated times for flexible and non‐flexible models. If the divergence times using both calibrations are plotted against each other, a linear relationship is obtained (see Supporting information Table S6 for linear regression). Calibration 2 did not seem to have a very strong effect on the estimated divergence times nor ΔT. For example, under a root prior of 3.5 ± 0.05 Ga and employing Calibration 2, ΔT was centered at 0.97 Ga (Table 4). We also tested the effect of removing the oldest calibration point from the analysis (point 11, Figure 3), this has the effect of making many nodes younger, yet ΔT remained in the range of 0.8 to 1.3 Ga depending on the level of flexibility allowed (Table 1).

In contrast, the choice of model for the evolution of substitution rates had a strong impact on the estimated divergence times as shown in Supporting information Figure S3 and Table S7. Supporting information Figure S3 presents a comparison of divergence time estimates of a tree calculated using a relaxed log‐normal autocorrelated molecular clock with a tree calculated using an uncorrelated gamma model on the rates of evolution. The autocorrelated model assumes that neighboring branches are more likely to evolve at a similar rate, while the uncorrelated model assumes that the rate of evolution of each branch can vary independently (Ho & Duchene, 2014; Lepage, Bryant, Philippe, & Lartillot, 2007). Under the uncorrelated model, the estimated divergence times of many nodes were aberrantly shifted to younger ages: for example, most cyanobacterial and eukaryotic D1 clustered in the range of 700 to 0 Ma, which is inconsistent with the fossil record. The molecular mechanism behind this difference could be related to the fact that photochemistry imposes a strong constraint on the evolution of reaction center proteins: as all of them must coordinate and maintain all redox cofactors, chlorophylls, quinones, carotenoids, and a non‐heme iron, at a precise orientation and distance from each other to allow for control of electron transfer rates and redox potentials. These rates and potentials are crucial not only for function but also for protection against the formation of reactive oxygen species (Cardona et al., 2012; Rutherford, Osyczka, & Rappaport, 2012). It seems reasonable then, that the rates of evolution of all Type II reaction center proteins should be similar between closely related groups, thus corresponding to an autocorrelated model.

## 3 DISCUSSION

### 3.1 Change in sequence identity as a function of time

As an approximation to the evolution of the core subunits of PSII, we plotted the level of amino acid sequence identity of D1 and D2, a simple measurement of phylogenetic distance, as a function of known divergence times (Figure 2). Two main conclusions can be derived from this plot independent of, yet in agreement with, the molecular clock analysis. Firstly, the three earliest stages in the evolution of oxygenic photosynthesis: the divergence of Type I and Type II reaction centers, the divergence of *anoxygenic* and *oxygenic* families of Type II reaction center proteins, and the divergence of D1 and D2, are more likely to have started soon after the origin of the first reaction centers rather than near the GOE. Taking into consideration that there is evidence for photosynthesis at 3.5 Ga (Butterfield, 2015; Nisbet & Fowler, 2014; Schopf, Kitajima, Spicuzza, Kudryavtsev, & Valley, 2018; Tice & Lowe, 2004), then all three stages could well predate this time. Secondly, the MRCA of Cyanobacteria is more likely to have lived near the time of the GOE rather than shortly after the origin of photosynthesis in the early Archean. This common ancestor must have had a standard PSII, which places it relatively far after the origin of photosynthesis. The early divergence of D1 and D2 means that the earliest stages in the evolution of oxygenic photosynthesis could predate the MRCA of Cyanobacteria by over a billion years.

### 3.2 Bayesian relaxed molecular clock analysis

The application of a Bayesian molecular clock analysis to the phylogeny of Type II reaction centers can be problematic because this was designed to deal with heterotachy of orthologs within and between lineages (Ho & Duchene, 2014) and thus they may not be able to perfectly model the variation in the rates of evolution across the long history of ancient paralogs. Therefore, the reader should interpret the reported age estimates as an approximation, as simulations of plausibility, and as a tool to distinguish between competing hypotheses. Even so, molecular clocks on duplicated proteins have been used informatively before (Aguileta, Bielawski, & Yang, 2006; Boyd et al., 2011; Gold, Caron, Fournier, & Summons, 2017; Sharma & Wheeler, 2014; Shih & Matzke, 2013) and our molecular clock is strongly constrained by three pieces of well‐supported and independent evidence: (a) by evidence of photosynthesis at 3.5 Ga, (b) by the fact that all Cyanobacteria and photosynthetic eukaryotes have inherited a standard form of D1, and (c) by the very slow rate of evolution of the core proteins over the Proterozoic. Under these constraints, the divergence between D1 and D2 is better explained by the duplication event occurring early in the evolutionary history of photosynthesis, in the early Archean, with the appearance of standard PSII occurring after a long period of evolutionary innovation. We highlight this long period by introducing the concept of ΔT. The magnitude of ΔT is dictated by the large phylogenetic distance between D1 and D2 and the slow rate of evolution determined from the geochemical and fossil record. Therefore, it is not surprising that under most models employed in this analysis, ΔT is in the range of 1.0 Ga.

**Figure 6:**
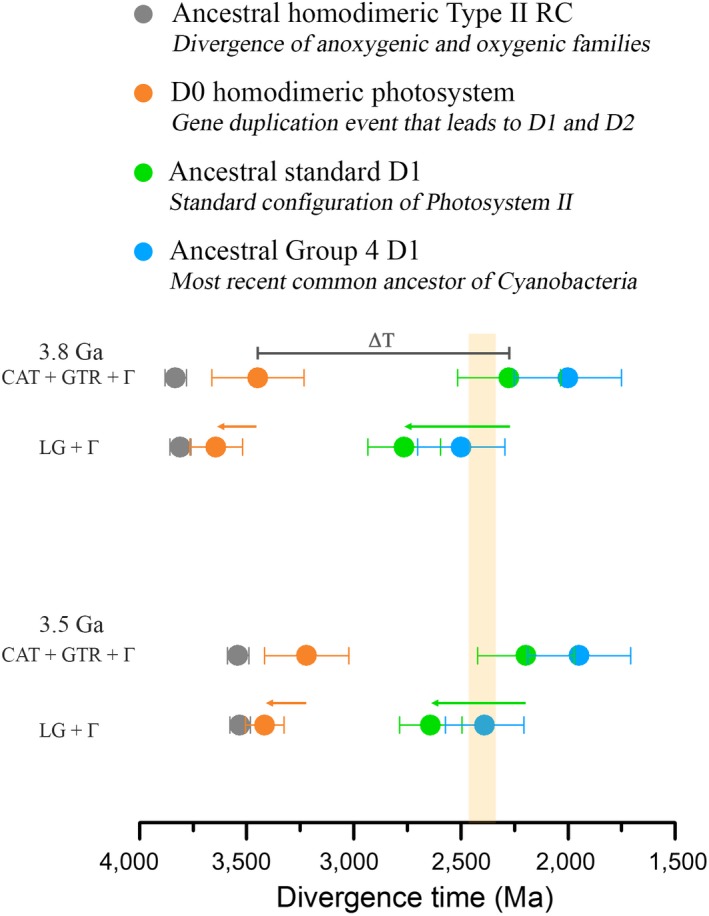
Scenarios for the evolution of Type II reaction centers. The rows of colored dots represent estimated divergence times of key nodes as highlighted in Figure 3 and calculated using the CAT + GTR + Γ and LG + Γ models and root priors of 3.8 or 3.5 ± 0.05 Ga. A highly oxidizing photosystem with enough power to split water is likely to have originated before the gene duplication event that led to D1 and D2 (orange dot). Making the MRCA of Cyanobacteria older (green arrow) pushes the earliest stages in the evolution of PSII and water oxidation closer to the origin of photosynthesis (orange arrow). The yellow vertical bar marks the GOE [Colour figure can be viewed at wileyonlinelibrary.com]

We have considered two possible evolutionary scenarios that are both consistent with a large ΔT (Figure 6). In the first scenario, the standard forms of D1 start to diverge at about 2.4 Ga, as seen in Figure 3, and diversify into G3 and G4 after the GOE. If we consider that the MRCA of Cyanobacteria had a G4 D1, this would set it after the GOE. This scenario, derived from the application of a relaxed molecular clock using a non‐parametric CAT model with flexible boundaries, is in agreement with the recent observations by Shih, Hemp et al. (2017) and other molecular clock studies that placed the divergence of *Gloeobacter* after the GOE (David & Alm, 2011; Feng, Cho, & Doolittle, 1997; Marin, Battistuzzi, Brown, & Hedges, 2017). In this scenario, assuming that the earliest events in the history of photosynthesis started about 3.5 Ga, the divergence of D1 and D2 is set at about 3.2 Ga.

In the second scenario, we considered that the MRCA of Cyanobacteria occurs before the GOE as suggested by other molecular clock analyses (Falcon et al., 2010; Sanchez‐Baracaldo, 2015; Schirrmeister et al., 2015). In the present work, this scenario can be fitted most satisfactorily with the application of a relaxed molecular clock using an empirical amino acid substitution model (LG + Γ). In this scenario, under a root prior of 3.5 Ga, the appearance of the ancestral standard form of D1 is set at about 2.6 Ga; and this has the consequence of pushing the divergence of D1 and D2 closer to the root, and thus D0 is set at about 3.4 Ga (Table 1 and 2, Figure 6). This effect is due to the fact that the phylogenetic distance between D1 and D2 is invariable, and thus under any scenario, the data are better explained by a long span of time separating D0 and the standard heterodimeric PSII. What can be concluded from this is that the older the MRCA of Cyanobacteria is, the more likely it is that the divergence of D1 and D2 started near the origin of photochemical reaction centers and thus, near the origin of photosynthesis.

Our results are consistent with the emerging view that most, if not all, identified groups of phototrophs started to diversify long after the origin of photosynthesis. Recent molecular clock analysis aimed at dating the MRCA of various groups of phototrophs have concluded that these appeared at around the GOE or after the GOE (Cardona, 2018; Magnabosco et al., 2018; Shih, Hemp et al., 2017; Shih, Ward et al., 2017). Our results support this view, yet at the same time they highlight the great antiquity of photosynthesis by showing that some of the early duplications of the core reaction center proteins likely predate the MRCA of each of the known groups of phototrophs by a large span of time. The implications of this emerging view are discussed in more detail in the Supporting information Discussion section “*Diversification of phototrophic lineages*.”

### 3.3 Rates of evolution

It should be observed that if a relatively constant rate of change were to be applied to the evolution of D1 and D2, the ancestral duplication would be placed long before the formation of the planet (dashed red line in Figure 2). Given the fact that the rate of evolution of D1 and D2 is constrained at slow rates from the Proterozoic until present times, the period of fast evolutionary change has to be located at the earliest stages to account for the evolution of photosynthesis within a reasonable timeframe. The rates calculated using the molecular clocks are in agreement with the observations based solely on a comparison of the level of sequence identity and revealed that even considering an origin of Type II reaction centers at 3.8 Ga the maximum rate during the evolution of D1 and D2, *ν*_max_, is more than thirty times greater than the measured rates since the Proterozoic. The much faster rates required to place the early stages of reaction center evolution (including the D0 duplication) in the Paleoarchean are difficult to reconcile with the structural complexity inherited by all known reaction centers. This structural complexity should have subjected the rates of evolution to strong constraints from early on. We hypothesize that such rates were only possible near the origin of reaction centers when life was still “figuring out” how to do photosynthesis for the first time.

There are other scenarios that can potentially account for the exponential decrease in the rates observed here, and these are further discussed in the Supporting information Discussion section “*Rates of Evolution*”: briefly, (a) duplication followed by neofunctionalization (Innan & Kondrashov, 2010; Lynch & Conery, 2000), (b) a proposed exponential decrease in the temperature‐dependent rate of deamination of cytosine on a warm early Earth (Lewis, Crayle, Zhou, Swanstrom, & Wolfenden, 2016), (c) greater exposure to UV radiation in the photic zone in the absence of an ozone layer (Cockell, 2000), and (d) a combination of the above.

### 3.4 Was there water oxidation before D1 and D2?

Even before the gene duplication that allowed the divergence of D1 and D2, the ancestral homodimeric photosystem had enough oxidizing power to form the neutral tyrosyl radical: high enough to surpass the E_*m*_ of water oxidation to oxygen. However, this does not necessarily imply that water oxidation was occurring at this time. Is there any evidence that would support or argue against an origin of water oxidation before the D1 and D2 duplication event?

Almost every major structural difference between anoxygenic Type II reaction center proteins and the core proteins of PSII can be explained in the context of water oxidation, protection against the formation of reactive oxygen species, and enhanced repair and assembly mechanisms due to oxidative damage from the formation of singlet oxygen around the photochemical pigments. A similar rationale has recently been proposed for the divergence of homodimeric Type I reaction centers of anoxygenic photosynthesis and heterodimeric Photosystem I of oxygenic photosynthesis (Orf, Gisriel, & Redding, 2018).

**Figure 7:**
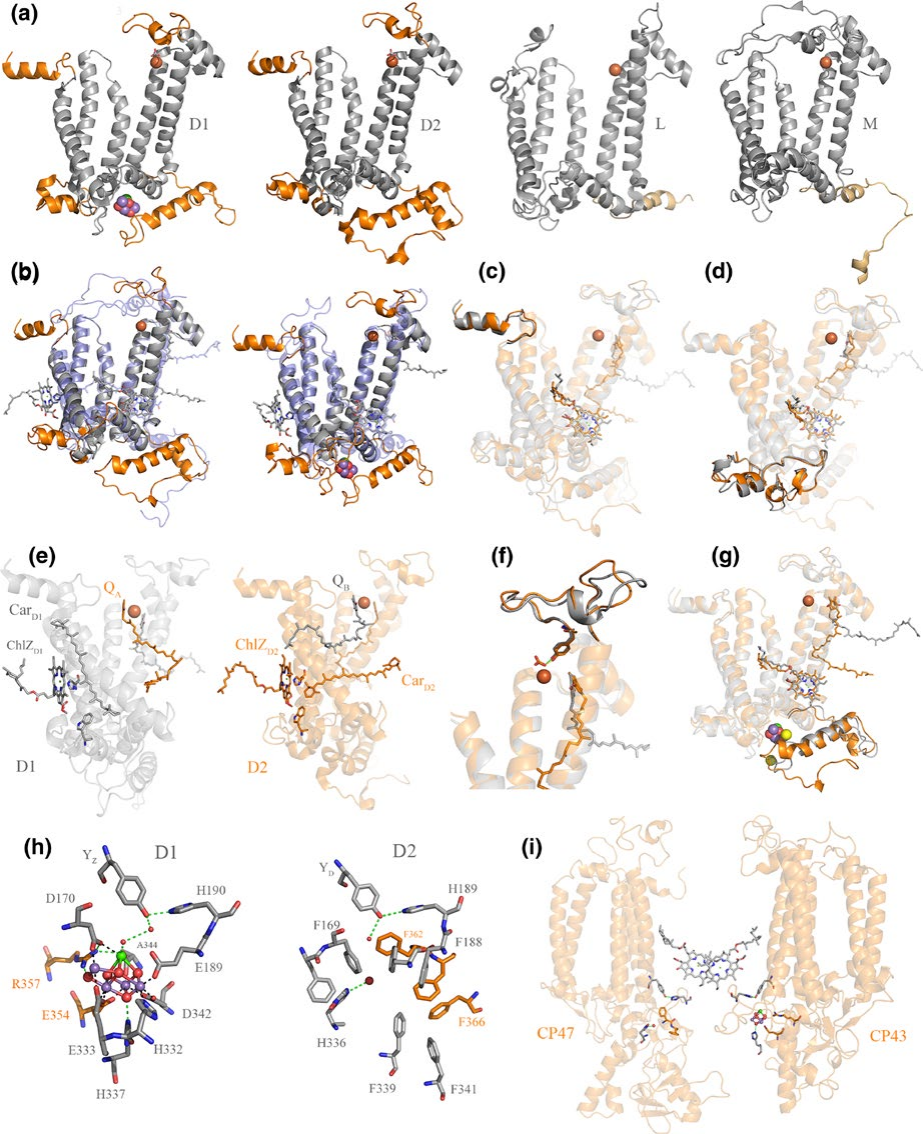
Structural comparisons of Type II reaction center proteins. (a) Several structural domains are conserved in D1 and D2 but are absent in L and M: These are highlighted in orange. D1 and D2 are plotted from the crystal structure of PSII from *Thermosynechococcus vulcanus*, PDB: 3WU2 (Umena et al., 2011) and L and M from *Thermochromatium tepidum*, PDB: 3WMM (Niwa et al., 2014). (b) Overlap of D2 (gray) with M (transparent blue) and D1 (gray) with L (transparent blue). (c) Overlap of D1 (gray) and D2 (orange) highlighting the conserved N‐terminus. (d) Overlap of D1 and D2 highlighting the conserved protein fold between the 1st and 2nd transmembrane helices. (e) D1 is shown in gray and D2 in orange. ChlZ_D__1_, Car_D1_, W105, and Q_B_ are shown in gray sticks. ChlZ_D__2_, Car_D2_, W104, and Q_A_ are shown in orange sticks. (f) Overlap of D1 and D2 highlighting the conserved protein fold where the bicarbonate binding site is placed. (g) Overlap of D1 and D2 highlighting the conserved protein fold at the C‐terminus. (h) The Mn_4_CaO_5_ cluster coordination sphere and the equivalent location in D2. (i) Perspective view showing the interaction of CP47 and CP43 with the electron donor side of D2 and D1, respectively. The structural homology of CP43 and CP47 indicates that these originated from a gene duplication event making the homodimeric antenna to a homodimeric core (Cardona, 2016). The reason why CP47, and in particular CP43, interact with the donor side of PSII is an unsolved mystery given the fact that their main role is that of light harvesting. It can be rationalized however if water oxidation started in a homodimeric reaction center early during the evolution of photosynthesis (Cardona, 2017) [Colour figure can be viewed at wileyonlinelibrary.com]

Five major structural differences distinguish D1 and D2 from L and M (Figure 7a and b). Starting from the N‐terminus:

#### 3.4.1 The N‐terminus itself (Figure 7c)

PSII is known to generate singlet oxygen, a very damaging form of reactive oxygen species that without control can lead to irreparable damage to the organism and death. Singlet oxygen is produced when molecular oxygen interacts with the excited triplet state of chlorophyll (Krieger‐Liszkay, Fufezan, & Trebst, 2008; Rutherford et al., 2012; Vass & Cser, 2009). Triplet chlorophyll is in turn formed when excess harvested light energy cannot be efficiently dissipated or when the forward electron transfer reactions of PSII are blocked and instead a backflow of electrons occurs (back‐reactions) (Krieger‐Liszkay et al., 2008; Santabarbara, Bordignon, Jennings, & Carbonera, 2002). Thus, the unavoidable production of singlet oxygen by PSII results in rapid damage of the core proteins in such a way that the half‐lifetime of D1 is about 30 min. D1 is known to be the protein with the fastest turnover in the photosynthetic membrane (Aro, Virgin, & Andersson, 1993). The half‐lifetime of D2 is also relatively fast, measured at about 3 hours. In comparison, the half‐lifetime of Photosystem I core proteins is about 2 days (Yao, Brune, & Vermaas, 2012). Damaged D1 and D2 are degraded by dedicated FtsH proteases, which target and recognize the N‐terminus of both subunits. Deletion of the N‐terminus results in impairment of degradation and repair (Komenda et al., 2007; Krynicka, Shao, Nixon, & Komenda, 2015). The preserved sequence and structural identity at the N‐terminus of both D1 and D2 suggests that the evolution of enhanced repair mechanisms had started to evolve before the duplication. Consistent with this, the evolution of all bacterial FtsH proteases confirms that the lineage of proteases specifically dedicated to the repair of PSII makes a monophyletic and deep‐branching clade (Shao, Cardona, & Nixon, 2018). As is the case for the evolution of reaction center proteins, this deep‐branching clade of PSII‐FtsH proteases appeared to have diverged before the radiation of those found in all the other groups of phototrophs (Shao et al., 2018).

#### 3.4.2 A protein fold between the 1st and 2nd transmembrane helices (Figure 7d)

In D1 and D2, this region is made of 54 and 52 residues in comparison with 28 and 35 residues in L and M, respectively. This fold is enlarged in D1 and D2 to provide a site for protein–protein interactions with the small peripheral subunits and the extrinsic polypeptides (Cardona, 2015, 2016), none of which are present in anoxygenic Type II reaction centers. In D1, this site provides a connection to PsbI, M, T, and O; and in D2 to the cytochrome *b*_559_, PsbH, J, and X. The small subunits are necessary to support a more complex assembly and disassembly cycle due to much higher rates of repair (Komenda, Sobotka, & Nixon, 2012). They provide stability, help with photoprotective functions, assist with the photoassembly of the Mn_4_CaO_5_ cluster (Dobakova, Tichy, & Komenda, 2007; Hamilton et al., 2014; Komenda, Lupinkova, & Kopecky, 2002; Popelkova & Yocum, 2011; Sugiura, Nakamura, Koyama, & Boussac, 2015), and even contribute to the highly oxidizing potential of PSII (Ishikita, Saenger, Biesiadka, Loll, & Knapp, 2006). The extrinsic polypeptides are fundamental for the stability of the Mn_4_CaO_5_ cluster, in particular PsbO, also known as the manganese stabilizing protein (De Las Rivas, Balsera, & Barber, 2004; Franzen, Hansson, & Andreasson, 1985; Roose, Frankel, Mummadisetti, & Bricker, 2016). That this site and its structural fold is conserved in D1 and D2 suggests that before their divergence the ancestral homodimeric photosystem had already achieved a high degree of structural complexity and was interacting with a number of auxiliary subunits in a way that it is not matched by anoxygenic Type II reaction centers. Because the role of the auxiliary subunits is the support of water oxidation and associated functions, this expansion of structural complexity can only start after the origin of water oxidation.

#### 3.4.3 The peripheral pigment pairs, ChlZ_D1_‐Car_D1_ and ChlZ_D2_‐Car_D2_ (Figure 7e)

**Figure 8:**
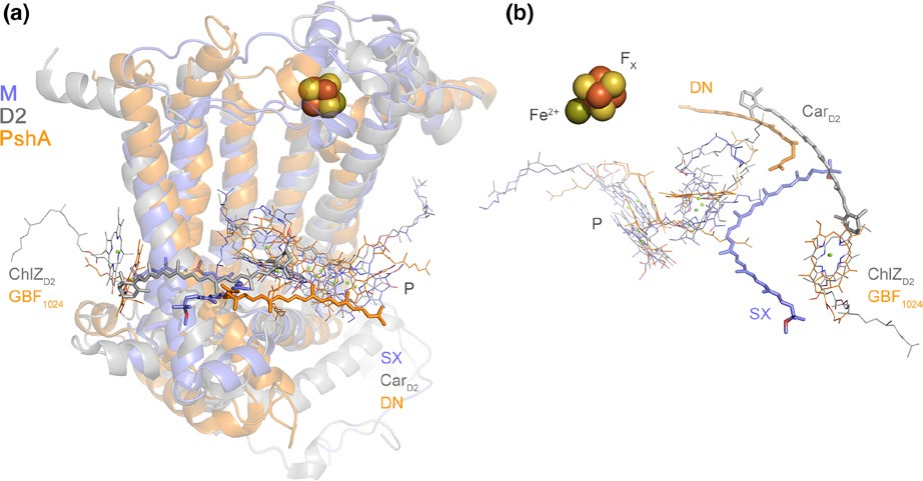
Carotenoids in the reaction center core. (a) Overlap of the M subunit of the anoxygenic Type II reaction center from *Thermochromatium tepidum* (light blue) with the D2 subunit of PSII from *Thermosynechococcus vulcanus* (gray) and the PshA subunit of the homodimeric Type I reaction center of *Heliobacterium modesticaldum* (orange), PDB ID: 5v8k (Gisriel et al., 2017). The protein backbone is showed in transparent ribbons, some of the photochemical pigments are displayed as thin sticks, and the closest carotenoids to the core are shown as thick sticks. SX stands for spirilloxanthin and DN for 4,4′‐diaponeurosporene. Car_D2_ is the core beta‐carotene bound by D2. Both SX and Car_D2_ have been demonstrated to have photoprotective roles, while the role of DN in the homodimeric Type I reaction center has not been demonstrated yet. However, based on its structural position overlapping with half of Car_D2_ and in Van der Waals contact with several bacteriochlorophyll *g* molecules, it can be predicted that it also has a photoprotective role. P denotes the photochemical “special pair” pigments. (b) A rotated view of the position of the carotenoids in the overlapped structures. The protein backbone has been hidden for clarity. ChlZ_D__2_ and the bacteriochlorophyll *g* molecule, GBF_1024_, occupy homologous positions [Colour figure can be viewed at wileyonlinelibrary.com]

D1 and D2 each coordinate a peripheral chlorophyll from a conserved histidine ligand in the 2nd transmembrane helix, known as ChlZ_D1_ and ChlZ_D2_. These peripheral pigments are absent in anoxygenic Type II reaction centers but are present in Type I reaction centers indicating that they existed before the divergence of D1 and D2 (Cardona, 2015). Both peripheral chlorophylls are required for photoautotrophic growth as mutations that impair their binding cannot assemble functional PSII (Lince & Vermaas, 1998; Ruffle et al., 2001). ChlZ_D1_ and ChlZ_D2_ are each in direct contact with a beta‐carotene molecule, known as Car_D1_ and Car_D2_ respectively, seen using crystallography first by Ferreira et al. (2004) and Loll, Kern, Saenger, Zouni, and Biesiadka (2005), but detected and characterized by spectroscopy well before that; see for example (Hanley, Deligiannakis, Pascal, Faller, & Rutherford, 1999; Kwa, Newell, van Grondelle, & Dekker, 1992; Noguchi, Mitsuka, & Inoue, 1994). The position of Car_D1_ and Car_D2_ differs in that the former is positioned perpendicular to the membrane plane while the latter is parallel to the membrane plane: However, one of the beta‐rings of each carotenoid links to ChlZ_D1_ and ChlZ_D2_ via strictly conserved tryptophan residues (D1‐W105 and D2‐W104, respectively), located in the unique protein fold between the 1st and 2nd transmembrane helices described above, and therefore absent in L and M. Car_D2_ is tilted relative to Car_D1_ partly to give way to the exchangeable plastoquinone, Q_B_. The core carotenoids of PSII have been shown to contribute little to light harvesting and have dominantly a protective role (Stamatakis, Tsimilli‐Michael, & Papageorgiou, 2014). The close association of ChlZ_D1_ and ChlZ_D2_ with carotenoids would suggest a role in protection, by quenching chlorophyll triplet states or directly scavenging singlet oxygen (Cogdell et al., 2000; Telfer, 2002). A role for the direct scavenging of singlet oxygen for both ChlZ_D1_‐Car_D1_ and ChlZ_D2_‐Car_D2_ has been suggested based on spectroscopy of isolated reaction centers (Telfer, Dhami, Bishop, Phillips, & Barber, 1994). Furthermore, ChlZ_D2_‐Car_D2_ have been demonstrated to be involved in protective electron transfer side pathways within PSII. For a detailed overview of these pathways see for example (Faller, Fufezan, & Rutherford, 2005). That ChlZ_D1_ and ChlZ_D2_ have been retained since before the divergence of Type I and Type II reaction centers indicates that they predate the D1 and D2 divergence. The acquisition of closely interacting carotenoids seems to have occurred therefore after the K and D0 divergence, but before the D1 and D2 split, in support of water oxidation before heterodimerization. However, carotenoids at a similar position to Car_D1_ and Car_D2_ have recently been identified in the homodimeric Type I reaction center of Heliobacteria (Gisriel et al., 2017) suggesting that these may predate the Type I/Type II split (Figure 8).

#### 3.4.4 An extended loop between the 4th and the 5th transmembrane helices (Figure 7f)

This is required for the coordination of bicarbonate, a ligand to the non‐heme iron (Ferreira et al., 2004), which is a distinctive feature of PSII. In anoxygenic Type II reaction centers, the non‐heme iron is coordinated asymmetrically by a glutamate from the M subunit. There is significant sequence and structural conservation of the bicarbonate binding site in D1 and D2. Two strictly conserved tyrosine residues D1‐Y246 and D2‐Y244 provide symmetric hydrogen bonds to bicarbonate (Ferreira et al., 2004). This indicates that bicarbonate binding was a feature existing before the divergence of D1 and D2. The role of bicarbonate had been a long‐standing mystery, but recently it was shown that binding and unbinding of bicarbonate modulates the E_*m*_ of the quinones, working as a switch from a productive state into a protective state that prevents chlorophyll triplet state and singlet oxygen formation (Brinkert, De Causmaecker, Krieger‐Liszkay, Fantuzzi, & Rutherford, 2016). Previously, G. N. Johnson, Rutherford, and Krieger (1995) had shown that a shift in the E_*m*_ of the fixed quinone, Q_A_, plays a key role in protection of PSII during assembly of the Mn_4_CaO_5_ cluster, a light‐driven process. It is understood now that such a shift is mediated by bicarbonate binding (Brinkert et al., 2016). A further conclusion from this is that the ancestral photosystem made of a D0 homodimer had already evolved to incorporate bicarbonate‐mediated protective mechanisms as well, implying oxygen evolution, and by extension, the assembly of a primordial water‐oxidizing complex.

#### 3.4.5 An extended C‐terminus and the Mn_4_CaO_5_ cluster binding site (Figure 7g)

D1 and D2 share an extended C‐terminus made of about 50 residues and with a distinctive alpha‐helix parallel to the membrane plane. The C‐terminus is necessary for the coordination of the Mn_4_CaO_5_ cluster, Cl^‐^ binding, water channels, and proton pathways (Debus, 2001; Linke & Ho, 2013; Umena, Kawakami, Shen, & Kamiya, 2011). Remnants of this C‐terminal extension may be found in some of the M and L subunits of phototrophic Proteobacteria and Chloroflexi (Cardona, 2015), but see Figure 7a. In D1 H332, E333, D342, and A344 coordinate the Mn_4_CaO_5_ cluster (Figure 7h). H337 provide a hydrogen bond to one of the bridging oxygens. In addition, E354 from the CP43 antenna subunit coordinates two of the Mn atoms and R357 offers a hydrogen bond to another bridging oxygen. While there is not a cluster in D2, an examination of the donor side in the immediate vicinity of the redox active tyrosine shows that the site has been blocked by a number of phenylalanine residues (Svensson, Vass, & Styring, 1991). Every ligand to the cluster is matched by a phenylalanine residue in D2 (Figure 7h). In the CP47 antenna, the ligands found in the CP43 are also replaced by phenylalanine residues (Figure 7h and i). The only exception is a histidine in a position equivalent to H337, which perhaps not coincidentally, provides a hydrogen bond to a water molecule locked within the hydrophobic patch made by the phenylalanine residues. No such phenylalanine patch is found in any other reaction center protein, except D2 (Cardona, 2016). The presence of a redox tyrosine in D2 and what seems like a vestigial metal‐binding site would be puzzling if the water‐oxidizing cluster evolved after the divergence of D1 and D2 in a heterodimeric system, but it would make sense if a primordial water‐oxidizing cluster appeared first on both sides of the reaction center in the ancestral homodimeric photosystem.

In conclusion, based on the above structural and functional evidence we find it highly likely that water oxidation originated before the divergence of D1 and D2. Hence, in a homodimeric Type II ancestor containing two identical exchangeable quinones charge separation would result in enhanced back‐reactions caused by electron transfer to a quinone site when empty or when occupied by a reduced form of the quinone; and additionally, by shorter back‐reaction pathways (Cardona et al., 2012; Rutherford et al., 2012). Back‐reactions would give rise to chlorophyll triplet states in the heart of the reaction center (Dutton, Leight, & Seibert, 1972; Rutherford, Paterson, & Mullet, 1981). In the evolution of a water‐splitting homodimeric ancestor with an increased oxidizing potential, as mentioned above, avoidance of photodamage could be a significant selective pressure for heterodimerization, as the enhanced back‐reactions intrinsic to the homodimeric Type II reaction centers would have become a major problem only if oxygen were present. In addition, two primordial clusters on each side of the reaction center would double the chance of forming back‐reacting intermediary states driving forward heterodimerization (Keren, Ohad, Rutherford, Drepper, & Krieger‐Liszkay, 2000). An inefficient homodimeric water‐splitting photosystem would have encountered this problem first and thus come under strong selection pressure toward heterodimerization at an early time. The results of the present work fit with this picture and indicate that this transitional water‐oxidizing homodimeric state was very short‐lived.

Our present finding that the duplication leading to L and M occurred significantly later in anoxygenic Type II reaction centers opens the possibility that oxygen could have also been a driving force in their heterodimerization process, since K would have encountered these selection pressures at a time when oxygen concentrations began to rise or fluctuate in localized environments during the late Archean (Bosak, Liang, Sim, & Petroff, 2009; Lyons, Reinhard, & Planavsky, 2014; Riding, Fralick, & Liang, 2014; Wang et al., 2018). Mirroring the evolution of Type II reaction centers, a molecular clock study on Type I reaction centers showed that the duplication event that led to the heterodimerization of the core of Photosystem I was also more likely to be the oldest node after the root (Cardona et al., 2012). This duplication event is widely accepted to have been an evolutionary adaptation to oxygenic photosynthesis (Ben‐Shem, Frolow, & Nelson, 2004; Hohmann‐Marriott & Blankenship, 2008; Rutherford et al., 2012) and was found to predate the earliest diversification event of anoxygenic Type I reaction centers (Cardona, 2018); namely, the divergence of the reaction center of Heliobacteria from that which gave rise to those in phototrophic Chlorobi and Acidobacteria.

It is rather remarkable that the anoxygenic Type II reaction center of phototrophic Proteobacteria contains an asymmetrically located carotenoid in contact with a core bacteriochlorophyll (Deisenhofer & Michel, 1989), see Figure 8. The role of this carotenoid is to quench bacteriochlorophyll triplet states to prevent the formation of singlet oxygen (Cogdell et al., 2000). A carotenoid with a similar position to Car_D1_ and Car_D2_ in PSII has been now discovered in the structure of a homodimeric Type I reaction center (Gisriel et al., 2017). In addition, light‐harvesting complexes in all anoxygenic photosynthetic bacteria contain carotenoids (e.g., chlorosome, LH1, LH2, B808‐866), which perform photoprotective roles (Kim, Li, Maresca, Bryant, & Savikhin, 2007; Melo, Frigaard, Matsuura, & Naqvi, 2000; Tsukatani, Romberger, Golbeck, & Bryant, 2012). As an extension of this, it does not seem unreasonable to think that even ancestral populations of anoxygenic phototrophic bacteria were under strong selective pressure by the threat of bacteriochlorophyll and chlorophyll triplet‐induced formation of reactive oxygen species.

If primordial forms of oxygenic photosynthesis appeared so early in the history of life, the bioavailability of oxygen should have left a mark on the evolution of other ancient molecular processes. We think that this is indeed the case and in the Supporting information Discussion section “*Peculiar oxygen anomalies,*” we compile a number of observations in the literature that are potentially consistent with the conclusions presented in this study.

## 4 FINAL REMARKS

The evolution of Type II reaction centers highlights the long history of oxygenic photosynthesis before the GOE. We show that the span of time between the gene duplication event that led to D1 and D2 and the appearance of standard PSII could have been in the order of a billion years. We argue that water oxidation is likely to have started before the divergence of D1 and D2. So what happened during this long period of time? If water oxidation originated in a simpler homodimeric photosystem in a completely anaerobic world, the large increase in the structural complexity of PSII, PSI, and associated light‐harvesting complexes had to occur alongside this trajectory. This includes the acquisition of many peripheral and extrinsic protein subunits and the heterodimerization of D1 and D2, CP43 and CP47, and PsaA and PsaB. At the same time, the thermodynamic coupling between both photosystems and the retuning of the entire electron transport chain and all electron carriers to increasingly oxidizing conditions also had to occur. This expansion in complexity had to be coupled in PSII with the evolution of highly organized assembly and repair processes. Thus, the first water‐oxidizing reaction centers may have been active only for brief amounts of time in the absence of efficient repair, or alternatively they may have been more energetically costly to maintain resulting in a decreased productivity. Greater water oxidation efficiency also needed the innovation of photoprotective mechanisms acting at different time scales spanning several orders of magnitude, like dissipatory recombination pathways, non‐photochemical quenching, or state‐transitions. Furthermore, the light reactions of photosynthesis had to be linked to carbon fixation and other downstream metabolic process. Signaling, feedback, and regulatory mechanisms had to be put in place to control photosynthesis under varying environmental conditions and across a diel cycle. Needless to say, all anaerobic reactions and processes inhibited by oxygen originally found in the earliest anaerobic water‐oxidizing ancestors had to be separated from oxygen production or readapted to work under aerobic conditions. The link to carbon fixation is of particular importance since CO_2_ levels in the atmosphere were higher than now (Nutman, Bennett, & Friend, 2017; Sheldon, 2006). However, limiting diffusion across boundary layers in the then prevalent mats and stromatolites would have restricted anoxygenic and early oxygenic phototrophs alike. The development of water oxidation would have opened up the way to faster photosynthetic rates, spurring on gross primary production rates, later in the Archean, with the concomitant need for increases in nitrogen fixation. In consequence, if water oxidation originated at an early stage during the evolutionary history of life other geological processes should have delayed the oxygenation of the planet until the Great Oxidation Event (Bindeman et al., 2018; Smit & Mezger, 2017).

## 5 MATERIALS AND METHODS

### 5.1 Phylogeny of Type II reaction centers

Sequences were retrieved from the RefSeq NCBI database using PSI‐BLAST restricted to Cyanobacteria, Proteobacteria, and Gemmatimonadetes. A total of 1703 complete sequences were downloaded and aligned using Clustal Omega employing ten combined guide trees and Hidden Markov Model iterations (Sievers et al., 2011). To confirm that the alignment conformed with known structures, the 3D structures of the D1, D2, L, and M, from the crystal structures 3WU2 (Umena et al., 2011) of PSII and 2PRC (Lancaster & Michel, 1997) of the anoxygenic Type II reaction center were overlapped using the CEalign (Jia, Dewey, Shindyalov, & Bourne, 2004) plug‐in for PyMOL (Molecular Graphics System, Version 1.5.0.4 Schrödinger, LLC) and structural homologous positions were cross‐checked with the alignment. Maximum likelihood (ML) phylogenetic analysis was performed using PhyML 3.1 (Guindon et al., 2010) using the LG model of amino acid substitution. The amino acid equilibrium frequencies, proportion of invariable sites, and across site rate variation were allowed to be determined by the software from the dataset. Tree search operations were set as the best from the Nearest Neighbor Interchange and Subtree Pruning and Regrafting method, with the tree topology optimized using five random starts. The ML tree using all sequences is shown in Figure 1 and it replicates earlier evolutionary studies of Type II reaction centers that used simpler methods and fewer sequences (Beanland, 1990; Cardona, 2015).

### 5.2 Change in sequence identity as a function of time

To get a better understanding of the evolutionary trends of D1 and D2 as a function of time, we compared the percentage of sequence identity of D1 and D2 from species of photosynthetic eukaryotes with known or approximate divergence times. In total, 23 pairs of sequences were compared and are listed in Supporting information Table 1. When two sequences were of different length, the longest was taken as 100%. Of these 23 pairs, the first 16 were based on the fossil calibrations recommended by Clarke et al. (2011) after their extensive review of the plant fossil record. Divergence times were taken as the average of the hard minimum and soft maximum fossil ages suggested by the authors. The last seven comparisons are approximate dates taken from the molecular clock analysis of the evolution of red algae by Yang et al. (2016). The plot of sequence identity vs. approximate divergence time was then fitted with a linear regression and the fitting parameters are shown in Supporting information Table S2.

### 5.3 Bayesian relaxed molecular clock and fossil calibrations

A total of 54 bacterial sequences spanning the entire diversity of Type II reaction centers were selected for Bayesian molecular clock analysis, including atypical and standard forms of D1, Alpha‐, Beta‐, Gammaproteobacteria, Chloroflexales*,* and *Gemmatimonas phototrophica*. Furthermore, 10 D1 and 10 D2 sequences from photosynthetic eukaryotes from taxa with a well characterized fossil record were added to allocate calibration points. The phylogeny of Type II reaction centers was cross‐calibrated on D1 and D2 as listed in Table 5 and calibration points were assigned as presented in Figure 3, red dots.

**Table 5:** Calibration points

Node	Event	Maximum (Ma)	Minimum (Ma)
1	*Arabidopsis‐Populus* divergence	127	82
2	Angiosperms	248	124
3	Gymnosperms	366	306
4	Land plants	–	475
5	Diatoms	–	190
6	Floridae	–	600
7	MRCA of red algae	–	1,200
8	Heterocystous Cyanobacteria	–	1,600
9	Pleurocapsales	–	1,700
10	Early‐branching multicellular Cyanobacteria	–	1,900
11	MRCA of Cyanobacteria	–	2,450/2,700

Dates for the *Arabidopsis*/*Populus* divergence, the divergence of the angiosperms (*Amborella*), gymnosperms (*Cycas*), and land plants (*Marchantia*) were implemented as suggested and discussed by Clarke et al. (2011), representing points 1 to 4 in Figure 3. Three ages from eukaryotic algae were used too: An age of 190 Ma was assigned to the divergence of *Phaeodactylum trichornutum* and *Thalassiosira pseudonana,* based on fossil Jurassic diatoms from the Lias Group, reviewed by Sims, Mann, and Medlin (2006). A minimum age of 600 Ma based on a Late Neoproterozoic Chinese multicellular red alga fossil (Xiao, Knoll, Yuan, & Pueschel, 2004) was assigned to the split between the diatom sequences and the sequences from *Porphyra purpurea*, as a conservative estimate for the divergence of complex red algae, which predates this time (Yang et al., 2016). The oldest calibration point in photosynthetic eukaryotes was assigned as a minimum age of 1.2 Ga to the divergence of the sequences from *Cyanidium caldarium* a unicellular early‐branching red algae. This was used as a conservative estimate for the origin of photosynthetic eukaryotes. The earliest widely accepted fossil of a photosynthetic eukaryote is that from a multicellular red algae, *Bangiomorpha* (Butterfield, 2000; Knoll, Worndle, & Kah, 2013), thought to be 1.0 Ga (Gibson et al., 2017). Recently described multicellular eukaryotic algae fossils have been reported at 1.6 Ga (Bengtson et al., 2017; Qu, Zhu, Whitehouse, Engdahl, & McLoughlin, 2018; Sallstedt et al., 2018) suggesting that the earliest photosynthetic eukaryotes might be older than that, which would be consistent with recent molecular clock analysis (Yang et al., 2016; Sanchez‐Baracaldo, Raven, Pisani, & Knoll, 2017).

Previously implemented cyanobacterial fossils were also used to calibrate the clock (Blank & Sanchez‐Baracaldo, 2010; Sanchez‐Baracaldo, 2015; Sanchez‐Baracaldo et al., 2017). A minimum age of 1.6 Ga was assigned to Nostocales because described akinetes of this age have been found in cherts from Siberia, China, and Australia (Golubic, Sergeev, & Knoll, 1995; Schirrmeister, Sanchez‐Baracaldo, & Wacey, 2016; Tomitani, Knoll, Cavanaugh, & Ohno, 2006). Pleurocapsales are characterized by multiple fissions during cell division and fossils retaining this morphology have been described at 1.7 Ga (Golubic & Lee, 1999; Sergeev, Gerasimenko, & Zavarzin, 2002). The earliest well‐assigned filamentous Cyanobacteria fossils of comparable size to those of the early‐branching *Pseudanabaena* have been reported at 1.9 Ga (Golubic & Lee, 1999; Sanchez‐Baracaldo et al., 2017; Schirrmeister et al., 2016; Sergeev et al., 2002), and this was assigned as a minimum age to the sequences from *Pseudanabaena biceps*.

The oldest calibration point, point 11, was selected to be the branching point of the D2 and the G4 D1 from *Gloeobacter violaceous*. This was set to be around the age for the GOE and was assigned as a minimum age of 2.45 Ga (Calibration 1) (Bekker et al., 2004). For comparison, a calibration of 2.7 Ga was also used (Calibration 2) to test the effect on the estimated divergence times of an older age for crown group Cyanobacteria. Geological evidence suggests that oxygen “whiffs” or “oases” could significantly predating the GOE (Havig et al., 2017; Lyons et al., 2014; Planavsky et al., 2014; Wang et al., 2018) so this scenario is not entirely implausible.

The calibration points on D1 were allocated on Group 4 because this type of D1 is the only one retained in all Cyanobacteria with PSII, it is the only type of D1 inherited by photosynthetic eukaryotes, and it is the main D1 used to catalyze water oxidation under most growth conditions: see Cardona et al. (2015) for a detailed analysis of the evolution of D1 proteins. It should be noted therefore that the duplications leading to all other forms of D1 occurred before the most recent common ancestor of Cyanobacteria (Cardona et al., 2015).

It is well accepted that a form photosynthesis had already evolved by 3.5 Ga, which is usually assumed to be anoxygenic. This is demonstrated by both sedimentological and isotopic evidence for photoautotrophic microbial communities recorded in Paleoarchean rocks (Butterfield, 2015; Nisbet & Fowler, 2014; Tice & Lowe, 2004). In addition, sedimentary rocks and banded iron formations from Isua, Greenland, hint at the presence of photosynthetic bacteria in the marine photic zone as early as 3.7–3.8 Ga (Czaja et al., 2013; Grassineau, Abell, Appel, Lowry, & Nisbet, 2006; Knoll, 2015; Rosing, 1999; Rosing & Frei, 2004; Schidlowski, 1988). Therefore, we used a root prior of 3.5 Ga as a conservative estimate for photoautotrophy based on photochemical reaction centers. Nevertheless, because it is not yet known exactly when photosynthesis originated for the first time we also tested the effect of varying the root prior from 3.2 to 4.1 Ga on the estimated divergence time under restrictive and flexible scenarios.

A Bayesian Markov chain Monte Carlo approach was used to calculate the estimated divergence times. We used Phylobayes 3.3f, which allows for the application of a relaxed log‐normal autocorrelated molecular clock under the CAT + GTR + Γ model (Lartillot & Philippe, 2004; Lartillot et al., 2009) necessary for the implementation of flexible boundaries on the calibration points (Yang & Rannala, 2006). To understand the effect of different evolutionary models on the age estimates we compared the CAT + GTR + Γ model with (a) a LG + Γ model that sets less flexible boundaries on the calibration points, (b) a CAT + Γ model assuming a uniform (Poisson) distribution of amino acid equilibrium frequencies, or (c) an uncorrelated gamma model where the rates of substitution can vary independently. The flexible bounds on the CAT + GTR + Γ model were set to allow for 2.5% tail probability falling outside each calibration boundary or 5% in the case of a single minimum boundary. All molecular clocks were computed using four discrete categories for the gamma distribution and four chains were run in parallel until convergence.

In this work, we define the period of time between the duplication event that led to the divergence of D1 and D2 and the appearance of the ancestral standard D1 as ΔT. This value is calculated as the subtraction of the mean age of the latter node (Figure 3, green dot) from the former's mean node age (Figure 3, D0, orange dot). The instant rates of evolution, which are necessary for the computation of divergence time from the phylogeny, were retrieved from the output files of Phylobayes. These rates are calculated by the software as described by the developers elsewhere (Kishino, Thorne, & Bruno, 2001; Lepage et al., 2007) and are expressed as amino acid changes per site per unit of time. The rate at node D0 was termed *ν*_max_ and a baseline rate of evolution during the Proterozoic was obtained as the average of all node rates in Group 4 D1 and D2 and denoted *ν*_min_. All sequence datasets and estimated divergence times for each node of each tree used in this analysis are provided in the Supporting information.

## References

[gbi12322-bib-0001] Aguileta, G. , Bielawski, J. P. , & Yang, Z. H. (2006). Evolutionary rate variation among vertebrate beta globin genes: Implications for dating gene family duplication events. Gene, 380(1), 21–29. 10.1016/j.gene.2006.04.019 16843621

[gbi12322-bib-0002] Aro, E. M. , Virgin, I. , & Andersson, B. (1993). Photoinhibition of Photosystem II. Inactivation, protein damage and turnover. Biochimica et Biophysica Acta (BBA)‐Bioenergetics, 1143(2), 113–134. 8318516 10.1016/0005-2728(93)90134-2

[gbi12322-bib-0003] Beanland, T. J. (1990). Evolutionary relationships between Q‐Type photosynthetic reaction centers ‐ Hypothesis‐testing using parsimony. Journal of Theoretical Biology, 145(4), 535–545. 10.1016/S0022-5193(05)80487-4 2246901

[gbi12322-bib-0004] Bekker, A. , Holland, H. D. , Wang, P. L. , Rumble, D. III , Stein, H. J. , Hannah, J. L. , & Beukes, N. J. (2004). Dating the rise of atmospheric oxygen. Nature, 427(6970), 117–120. 10.1038/nature02260 14712267

[gbi12322-bib-0005] Bengtson, S. , Sallstedt, T. , Belivanova, V. , & Whitehouse, M. (2017). Three‐dimensional preservation of cellular and subcellular structures suggests 1.6 billion‐year‐old crown‐group red algae. PLOS Biology, 15(3), e2000735 10.1371/journal.pbio.2000735 28291791 PMC5349422

[gbi12322-bib-0006] Ben‐Shem, A. , Frolow, F. , & Nelson, N. (2004). Evolution of photosystem I ‐ from symmetry through pseudosymmetry to asymmetry. FEBS Letters, 564(3), 274–280. 10.1016/S0014-5793(04)00360-6 15111109

[gbi12322-bib-0007] Betts, H. C. , Puttick, M. N. , Clark, J. W. , Williams, T. A. , Donoghue, P. C. J. , & Pisani, D. (2018). Integrated genomic and fossil evidence illuminates life's early evolution and eukaryote origin. Nature Ecology & Evolution, 2, 1556–1562. 10.1038/s41559-018-0644-x 30127539 PMC6152910

[gbi12322-bib-0008] Bindeman, I. N. , Zakharov, D. O. , Palandri, J. , Greber, N. D. , Dauphas, N. , Retallack, G. J. , … Bekker, A. (2018). Rapid emergence of subaerial landmasses and onset of a modern hydrologic cycle 2.5 billion years ago. Nature, 557, 545–548. 10.1038/s41559-018-0644-x 29795252

[gbi12322-bib-0009] Blank, C. E. , & Sanchez‐Baracaldo, P. (2010). Timing of morphological and ecological innovations in the cyanobacteria ‐ a key to understanding the rise in atmospheric oxygen. Geobiology, 8(1), 1–23. 10.1111/j.1472-4669.2009.00220.x 19863595

[gbi12322-bib-0010] Blankenship, R. E. (1992). Origin and early evolution of photosynthesis. Photosynthesis Research, 33, 91–111. 11538390

[gbi12322-bib-0011] Bosak, T. , Liang, B. , Sim, M. S. , & Petroff, A. P. (2009). Morphological record of oxygenic photosynthesis in conical stromatolites. Proceedings of the National Academy of Sciences of USA, 106(27), 10939–10943. 10.1073/pnas.0900885106 PMC2708726 19564621

[gbi12322-bib-0012] Boyd, E. S. , Anbar, A. D. , Miller, S. , Hamilton, T. L. , Lavin, M. , & Peters, J. W. (2011). A late methanogen origin for molybdenum‐dependent nitrogenase. Geobiology, 9(3), 221–232. 10.1111/j.1472-4669.2011.00278.x 21504537

[gbi12322-bib-0013] Brinkert, K. , De Causmaecker, S. , Krieger‐Liszkay, A. , Fantuzzi, A. , & Rutherford, A. W. (2016). Bicarbonate‐induced redox tuning in Photosystem II for regulation and protection. Proceedings of the National Academy of Sciences of USA, 113(43), 12144–12149. 10.1073/pnas.1608862113 PMC5087022 27791001

[gbi12322-bib-0014] Bukh, J. , Pietschmann, T. , Lohmann, V. , Krieger, N. , Faulk, K. , Engle, R. E. , & Bartenschlager, R. (2002). Mutations that permit efficient replication of hepatitis C virus RNA in Huh‐7 cells prevent productive replication in chimpanzees. Proceedings of the National Academy of Sciences of USA, 99(22), 14416–14421. 10.1073/pnas.212532699 PMC137898 12391335

[gbi12322-bib-0015] Butterfield, N. J. (2000). *Bangiomorpha pubescens* n. gen., n. sp.: Implications for the evolution of sex, multicellularity, and the Mesoproterozoic/Neoproterozoic radiation of eukaryotes. Paleobiology, 26(3), 386–404. 10.1666/0094-8373(2000)026<0386:bpngns>2.0.co;2

[gbi12322-bib-0016] Butterfield, N. J. (2015). Proterozoic photosynthesis ‐ a critical review. Palaeontology, 58(6), 953–972. 10.1111/pala.12211

[gbi12322-bib-0017] Canestro, C. , Albalat, R. , Hjelmqvist, L. , Godoy, L. , Jornvall, H. , & Gonzalez‐Duarte, R. (2002). Ascidian and amphioxus adh genes correlate functional and molecular features of the ADH family expansion during vertebrate evolution. Journal of Molecular Evolution, 54(1), 81–89. 10.1007/s00239-001-0020-2 11734901

[gbi12322-bib-0018] Cardona, T. (2015). A fresh look at the evolution and diversification of photochemical reaction centers. Photosynthesis Research, 126(1), 111–134. 10.1007/s11120-014-0065-x 25512103 PMC4582080

[gbi12322-bib-0019] Cardona, T. (2016). Reconstructing the origin of oxygenic photosynthesis: Do assembly and photoactivation recapitulate evolution? Frontiers in Plant Science, 7, 257 10.3389/fpls.2016.00257 26973693 PMC4773611

[gbi12322-bib-0020] Cardona, T. (2017). Photosystem II is a chimera of reaction centers. Journal of Molecular Evolution, 84(2–3), 149–151. 10.1007/s00239-017-9784-x 28224181

[gbi12322-bib-0021] Cardona, T. (2018). Early Archean origin of heterodimeric Photosystem I. Heliyon, 4(3), e00548 10.1016/j.heliyon.2018.e00548 29560463 PMC5857716

[gbi12322-bib-0022] Cardona, T. , Murray, J. W. , & Rutherford, A. W. (2015). Origin and evolution of water oxidation before the last common ancestor of the Cyanobacteria. Molecular Biology and Evolution, 32(5), 1310–1328. 10.1093/molbev/msv024 25657330 PMC4408414

[gbi12322-bib-0023] Cardona, T. , Sedoud, A. , Cox, N. , & Rutherford, A. W. (2012). Charge separation in Photosystem II: A comparative and evolutionary overview. Biochimica et Biophysica Acta (BBA)‐Bioenergetics, 1817(1), 26–43. 10.1016/j.bbabio.2011.07.012 21835158

[gbi12322-bib-0024] Carrat, F. , & Flahault, A. (2007). Influenza vaccine: The challenge of antigenic drift. Vaccine, 25(39–40), 6852–6862. 10.1016/j.vaccine.2007.07.027 17719149

[gbi12322-bib-0025] Clarke, J. T. , Warnock, R. C. M. , & Donoghue, P. C. J. (2011). Establishing a time‐scale for plant evolution. New Phytologist, 192(1), 266–301. 10.1111/j.1469-8137.2011.03794.x 21729086

[gbi12322-bib-0026] Cockell, C. S. (2000). Ultraviolet radiation and the photobiology of earth's early oceans. Origins of Life and Evolution of Biospheres, 30(5), 467–499. 10.1023/a:1006765405786 11002893

[gbi12322-bib-0027] Cogdell, R. J. , Howard, T. D. , Bittl, R. , Schlodder, E. , Geisenheimer, I. , & Lubitz, W. (2000). How carotenoids protect bacterial photosynthesis. Philosophical Transactions of the Royal Society B‐Biological Sciences, 355(1402), 1345–1349. 10.1098/rstb.2000.0696 PMC1692869 11127989

[gbi12322-bib-0028] Crowe, S. A. , Dossing, L. N. , Beukes, N. J. , Bau, M. , Kruger, S. J. , Frei, R. , & Canfield, D. E. (2013). Atmospheric oxygenation three billion years ago. Nature, 501(7468), 535–538. 10.1038/nature12426 24067713

[gbi12322-bib-0029] Czaja, A. D. , Johnson, C. M. , Beard, B. L. , Roden, E. E. , Li, W. Q. , & Moorbath, S. (2013). Biological Fe oxidation controlled deposition of banded iron formation in the ca. 3770 Ma Isua Supracrustal Belt (West Greenland). Earth and Planetary Science Letters, 363, 192–203. 10.1016/j.epsl.2012.12.025

[gbi12322-bib-0030] Dau, H. , & Zaharieva, I. (2009). Principles, efficiency, and blueprint character of solar‐energy conversion in photosynthetic water oxidation. Accounts of Chemical Research, 42(12), 1861–1870. 10.1021/ar900225y 19908828

[gbi12322-bib-0031] David, L. A. , & Alm, E. J. (2011). Rapid evolutionary innovation during an Archaean genetic expansion. Nature, 469(7328), 93–96. 10.1038/Nature09649 21170026

[gbi12322-bib-0032] De Las Rivas, J. , Balsera, M. , & Barber, J. (2004). Evolution of oxygenic photosynthesis: Genome‐wide analysis of the OEC extrinsic proteins. Trends in Plant Science, 9(1), 18–25. 10.1016/j.tplants.2003.11.007 14729215

[gbi12322-bib-0033] Debus, R. J. (2001). Amino acid residues that modulate the properties of tyrosine Y‐Z and the manganese cluster in the water oxidizing complex of Photosystem II. Biochimica et Biophysica Acta (BBA)‐Bioenergetics, 1503, 164–186. 10.1016/s0005-2728(00)00221-8 11115632

[gbi12322-bib-0034] DeFelippis, M. R. , Murthy, C. P. , Broitman, F. , Weinraub, D. , Faraggi, M. , & Klapper, M. H. (1991). Electrochemical properties of tyrosine phenoxy and tryptophan indolyl radicals in peptides and amino‐acid‐analogs. Journal of Physical Chemistry, 95(8), 3416–3419. 10.1021/j100161a081

[gbi12322-bib-0035] DeFelippis, M. R. , Murthy, C. P. , Faraggi, M. , & Klapper, M. H. (1989). Pulse radiolytic measurement of redox potentials: The tyrosine and tryptophan radicals. Biochemistry, 28(11), 4847–4853. 2765513 10.1021/bi00437a049

[gbi12322-bib-0036] Deisenhofer, J. , & Michel, H. (1989). Nobel lecture. The photosynthetic reaction centre from the purple bacterium Rhodopseudomonas viridis. Embo Journal, 8(8), 2149–2170. 2676514 10.1002/j.1460-2075.1989.tb08338.x PMC401143

[gbi12322-bib-0037] Dobakova, M. , Tichy, M. , & Komenda, J. (2007). Role of the PsbI protein in Photosystem II assembly and repair in the cyanobacterium *Synechocystis* sp. PCC 6803. Plant Physiology, 145(4), 1681–1691. 10.1104/pp.107.107805 17921338 PMC2151680

[gbi12322-bib-0038] Duda, T. F. Jr , & Palumbi, S. R. (1999). Molecular genetics of ecological diversification: Duplication and rapid evolution of toxin genes of the venomous gastropod Conus. Proceedings of the National Academy of Sciences of USA, 96(12), 6820–6823. 10.1073/pnas.96.12.6820 PMC21999 10359796

[gbi12322-bib-0039] Dutton, P. L. , Leight, J. S. , & Seibert, M. (1972). Primary processes in photosynthesis: In situ ESR studies on the light induced oxidized and triplet state of reaction center bacteriochlorophyll. Biochemical and Biophysical Research Communications, 46(2), 406–413. 4333414 10.1016/s0006-291x(72)80153-0

[gbi12322-bib-0040] Falcon, L. I. , Magallon, S. , & Castillo, A. (2010). Dating the cyanobacterial ancestor of the chloroplast. ISME Journal, 4(6), 777–783. 10.1038/ismej.2010.2 20200567

[gbi12322-bib-0041] Faller, P. , Fufezan, C. , & Rutherford, A. W. (2005). Side‐path electron donors: Cytochrome b559, chlorophyll Z, and beta‐carotene In Wydrzynski T. J. & Satoh K. (Eds.), Photosystem II: The light‐driven water:Plastoquinone oxidoreductase (Vol. 22, pp. 347–365). Dordrecht, the Netherlands: Springer.

[gbi12322-bib-0042] Feng, D. F. , Cho, G. , & Doolittle, R. F. (1997). Determining divergence times with a protein clock: Update and reevaluation. Proceedings of the National Academy of Sciences of USA, 94(24), 13028–13033. 10.1073/pnas.94.24.13028 PMC24257 9371794

[gbi12322-bib-0043] Ferreira, K. N. , Iverson, T. M. , Maghlaoui, K. , Barber, J. , & Iwata, S. (2004). Architecture of the photosynthetic oxygen‐evolving center. Science, 303(5665), 1831–1838. 10.1126/science.1093087 14764885

[gbi12322-bib-0044] Franzen, L. G. , Hansson, O. , & Andreasson, L. E. (1985). The roles of the extrinsic subunits in Photosystem II as revealed by Electron Paramagnetic Resonance. Biochimica et Biophysica Acta (BBA)‐Bioenergetics, 808(1), 171–179. 10.1016/0005-2728(85)90040-4

[gbi12322-bib-0045] Frei, R. , Crowe, S. A. , Bau, M. , Polat, A. , Fowle, D. A. , & Døssing, L. N. (2016). Oxidative elemental cycling under the low O_2_ Eoarchean atmosphere. Scientific Reports, 6, 21058 10.1038/srep21058 26864443 PMC4750038

[gbi12322-bib-0046] Georgelis, N. , Braun, E. L. , & Hannah, L. C. (2008). Duplications and functional divergence of ADP‐glucose pyrophosphorylase genes in plants. BMC Evolutionary Biology, 8, 232 10.1186/1471-2148-8-232 18700010 PMC2529307

[gbi12322-bib-0047] Gibson, T. M. , Shih, P. M. , Cumming, V. M. , Fischer, W. W. , Crockford, P. W. , Hodgskiss, M. S. W. , & Halverson, G. P. (2017). Precise age of *Bangiomorpha pubescens* dates the origin of eukaryotic photosynthesis. Geology, 46(2), 135–138. 10.1130/G39829.1

[gbi12322-bib-0048] Gisriel, C. , Sarrou, I. , Ferlez, B. , Golbeck, J. H. , Redding, K. E. , & Fromme, R. (2017). Structure of a symmetric photosynthetic reaction center‐photosystem. Science, 357(6355), 1021–1025. 10.1126/science.aan5611 28751471

[gbi12322-bib-0049] Gold, D. A. , Caron, A. , Fournier, G. P. , & Summons, R. E. (2017). Paleoproterozoic sterol biosynthesis and the rise of oxygen. Nature, 543(7645), 420–423. 10.1038/nature21412 28264195

[gbi12322-bib-0050] Golubic, S. , & Lee, S. J. (1999). Early cyanobacterial fossil record: Preservation, palaeoenvironments and identification. European Journal of Phycology, 34(4), 339–348. 10.1017/S0967026299002279

[gbi12322-bib-0051] Golubic, S. , Sergeev, V. N. , & Knoll, A. H. (1995). Mesoproterozoic Archaeoellipsoides: Akinetes of heterocystous cyanobacteria. Lethaia, 28(4), 285–298. 10.1111/j.1502-3931.1995.tb01817.x 11539549

[gbi12322-bib-0052] Grassineau, N. V. , Abell, P. I. , Appel, P. W. U. , Lowry, D. , & Nisbet, E. G. (2006). Early life signatures in sulfur and carbon isotopes from Isua, Barberton, Wabigoon (Steep Rock), and Belingwe Greenstone Belts (3.8 to 2.7 Ga). Memoirs ‐ Geological Society of America, 198, 33–52. 10.1130/2006.1198(02)

[gbi12322-bib-0053] Guindon, S. , Dufayard, J. F. , Lefort, V. , Anisimova, M. , Hordijk, W. , & Gascuel, O. (2010). New algorithms and methods to estimate maximum‐likelihood phylogenies: Assessing the performance of PhyML 3.0. Systematic Biology, 59(3), 307–321. 10.1093/sysbio/syq010 20525638

[gbi12322-bib-0054] Hamilton, M. L. , Franco, E. , Deak, Z. , Schlodder, E. , Vass, I. , & Nixon, P. J. (2014). Investigating the photoprotective role of Cytochrome b‐559 in Photosystem II in a mutant with altered ligation of the haem. Plant and Cell Physiology, 55(7), 1276–1285. 10.1093/pcp/pcu070 24850839

[gbi12322-bib-0055] Hanley, J. , Deligiannakis, Y. , Pascal, A. , Faller, P. , & Rutherford, A. W. (1999). Carotenoid oxidation in Photosystem II. Biochemistry, 38(26), 8189–8195. 10.1021/bi990633u 10387064

[gbi12322-bib-0056] Havig, J. R. , Hamilton, T. L. , Bachan, A. , & Kump, L. R. (2017). Sulfur and carbon isotopic evidence for metabolic pathway evolution and a four‐stepped Earth system progression across the Archean and Paleoproterozoic. Earth‐Science Reviews, 174, 1–21. 10.1016/j.earscirev.2017.06.014

[gbi12322-bib-0057] Ho, S. Y. W. , & Duchene, S. (2014). Molecular‐clock methods for estimating evolutionary rates and timescales. Molecular Ecology, 23(24), 5947–5965. 10.1111/mec.12953 25290107

[gbi12322-bib-0058] Ho, M. Y. , Shen, G. , Canniffe, D. P. , Zhao, C. , & Bryant, D. A. (2016). Light‐dependent chlorophyll f synthase is a highly divergent paralog of PsbA of Photosystem II. Science, 353(6302), aaf9178 10.1126/science.aaf9178 27386923

[gbi12322-bib-0059] Hohmann‐Marriott, M. F. , & Blankenship, R. E. (2008). Anoxygenic Type‐I photosystems and evolution of photosynthetic reaction centers In Fromme P. (Ed.), Photosynthetic protein complexes (pp. 295–324): Weinheim, Germany: Wiley‐VCH Verlag GmbH & Co. KGaA.

[gbi12322-bib-0060] Hughes, A. L. (1997). Rapid evolution of immunoglobulin superfamily C2 domains expressed in immune system cells. Molecular Biology and Evolution, 14(1), 1–5. 9000748 10.1093/oxfordjournals.molbev.a025694

[gbi12322-bib-0061] Innan, H. , & Kondrashov, F. (2010). The evolution of gene duplications: Classifying and distinguishing between models. Nature Reviews Genetics, 11(2), 97–108. 10.1038/nrg2689 20051986

[gbi12322-bib-0062] Ishikita, H. , Saenger, W. , Biesiadka, J. , Loll, B. , & Knapp, E. W. (2006). How photosynthetic reaction centers control oxidation power in chlorophyll pairs P680, P700, and P870. Proceedings of the National Academy of Sciences of USA, 103(26), 9855–9860. 10.1073/pnas.0601446103 PMC1502543 16788069

[gbi12322-bib-0063] Jia, Y. T. , Dewey, G. , Shindyalov, I. N. , & Bourne, P. E. (2004). A new scoring function and associated statistical significance for structure alignment by CE. Journal of Computational Biology, 11(5), 787–799. 10.1089/1066527042432260 15700402

[gbi12322-bib-0064] Johnson, G. N. , Rutherford, A. W. , & Krieger, A. (1995). A change in the midpoint potential of the quinone Q(a) in Photosystem II associated with photoactivation of oxygen evolution. Biochimica et Biophysica Acta (BBA)‐Bioenergetics, 1229(2), 202–207. 10.1016/0005-2728(95)00003-2

[gbi12322-bib-0065] Johnson, J. E. , Webb, S. M. , Thomas, K. , Ono, S. , Kirschvink, J. L. , & Fischer, W. W. (2013). Manganese‐oxidizing photosynthesis before the rise of cyanobacteria. Proceedings of the National Academy of Sciences of USA, 110(28), 11238–11243. 10.1073/pnas.1305530110 PMC3710856 23798417

[gbi12322-bib-0066] Kagan, R. M. , McFadden, H. J. , McFadden, P. N. , O'Connor, C. , & Clarke, S. (1997). Molecular phylogenetics of a protein repair methyltransferase. Comparative Biochemistry and Physiology B, 117(3), 379–385. 10.1016/S0305-0491(96)00333-1 9253175

[gbi12322-bib-0067] Kay, E. H. , & Hoekstra, H. E. (2008). Rodents. Current Biology, 18(10), R406–R410. 10.1016/j.cub.2008.03.019 18492466

[gbi12322-bib-0068] Keren, N. , Ohad, I. , Rutherford, A. W. , Drepper, F. , & Krieger‐Liszkay, A. (2000). Inhibition of Photosystem II activity by saturating single turnover flashes in calcium‐depleted and active Photosystem II. Photosynthesis Research, 63(3), 209–216. 10.1023/A:1006435530817 16228431

[gbi12322-bib-0069] Kim, H. , Li, H. , Maresca, J. A. , Bryant, D. A. , & Savikhin, S. (2007). Triplet exciton formation as a novel photoprotection mechanism in chlorosomes of *Chlorobium tepidum* . Biophysical Journal, 93(1), 192–201. 10.1529/biophysj.106.103556 17434948 PMC1914439

[gbi12322-bib-0070] Kishino, H. , Thorne, J. L. , & Bruno, W. J. (2001). Performance of a divergence time estimation method under a probabilistic model of rate evolution. Molecular Biology and Evolution, 18(3), 352–361. 10.1093/oxfordjournals.molbev.a003811 11230536

[gbi12322-bib-0071] Knoll, A. H. (2015). Paleobiological perspectives on early microbial evolution. Cold Spring Harbor Perspectives in Biology, 7(7), a018093 10.1101/cshperspect.a018093 26134315 PMC4484972

[gbi12322-bib-0072] Knoll, A. H. , Worndle, S. , & Kah, L. C. (2013). Covariance of microfossil assemblages and microbialite textures across an upper mesoproterozoic carbonate platform. Palaios, 28(7–8), 453–470. 10.2110/palo.2013.p13-005r

[gbi12322-bib-0073] Komenda, J. , Lupinkova, L. , & Kopecky, J. (2002). Absence of the psbH gene product destabilizes Photosystem II complex and bicarbonate binding on its acceptor side in *Synechocystis* PCC 6803. European Journal of Biochemistry, 269(2), 610–619. 11856320 10.1046/j.0014-2956.2001.02693.x

[gbi12322-bib-0074] Komenda, J. , Sobotka, R. , & Nixon, P. J. (2012). Assembling and maintaining the Photosystem II complex in chloroplasts and cyanobacteria. Current Opinion in Plant Biology, 15(3), 245–251. 10.1016/j.pbi.2012.01.017 22386092

[gbi12322-bib-0075] Komenda, J. , Tichy, M. , Prasil, O. , Knoppova, J. , Kuvikova, S. , de Vries, R. , & Nixon, P. J. (2007). The exposed N‐terminal tail of the D1 subunit is required for rapid D1 degradation during Photosystem II repair in *Synechocystis* sp PCC 6803. Plant Cell, 19(9), 2839–2854. 10.1105/tpc.107.053868 17905897 PMC2048700

[gbi12322-bib-0076] Krieger‐Liszkay, A. , Fufezan, C. , & Trebst, A. (2008). Singlet oxygen production in Photosystem II and related protection mechanism. Photosynthesis Research, 98(1–3), 551–564. 10.1007/s11120-008-9349-3 18780159

[gbi12322-bib-0077] Krynicka, V. , Shao, S. , Nixon, P. J. , & Komenda, J. (2015). Accessibility controls selective degradation of Photosystem II subunits by FtsH protease. Nature Plants, 1, 15168 10.1038/nplants.2015.168 27251713

[gbi12322-bib-0078] Kwa, S. L. S. , Newell, W. R. , van Grondelle, R. , & Dekker, J. P. (1992). The reaction center of Photosystem II studied with polarized fluorescence spectroscopy. Biochimica et Biophysica Acta (BBA)‐Bioenergetics, 1099(3), 193–202. 10.1016/0005-2728(92)90027-y

[gbi12322-bib-0079] Lancaster, C. R. D. , & Michel, H. (1997). The coupling of light‐induced electron transfer and proton uptake as derived from crystal structures of reaction centres from *Rhodopseudomonas viridis* modified at the binding site of the secondary quinone, Q(B). Structure, 5(10), 1339–1359. 10.1016/S0969-2126(97)00285-2 9351808

[gbi12322-bib-0080] Lartillot, N. , Lepage, T. , & Blanquart, S. (2009). PhyloBayes 3: A Bayesian software package for phylogenetic reconstruction and molecular dating. Bioinformatics, 25(17), 2286–2288. 10.1093/bioinformatics/btp368 19535536

[gbi12322-bib-0081] Lartillot, N. , & Philippe, H. (2004). A Bayesian mixture model for across‐site heterogeneities in the amino‐acid replacement process. Molecular Biology and Evolution, 21(6), 1095–1109. 10.1093/molbev/msh112 15014145

[gbi12322-bib-0082] Lepage, T. , Bryant, D. , Philippe, H. , & Lartillot, N. (2007). A general comparison of relaxed molecular clock models. Molecular Biology and Evolution, 24(12), 2669–2680. 10.1093/molbev/msm193 17890241

[gbi12322-bib-0083] Lewis, C. A. , Crayle, J. , Zhou, S. T. , Swanstrom, R. , & Wolfenden, R. (2016). Cytosine deamination and the precipitous decline of spontaneous mutation during Earth's history. Proceedings of the National Academy of Sciences of USA, 113(29), 8194–8199. 10.1073/pnas.1607580113 PMC4961170 27382162

[gbi12322-bib-0084] Lince, M. T. , & Vermaas, W. (1998). Association of His117 in the D2 protein of Photosystem II with a chlorophyll that affects excitation‐energy transfer efficiency to the reaction center. European Journal of Biochemistry, 256(3), 595–602. 10.1046/j.1432-1327.1998.2560595.x 9780236

[gbi12322-bib-0085] Linke, K. , & Ho, F. M. (2013). Water in Photosystem II: Structural, functional and mechanistic considerations. Biochimica et Biophysica Acta (BBA)‐Bioenergetics, 1837(1), 14–32. 10.1016/j.bbabio.2013.08.003 23978393

[gbi12322-bib-0086] Loll, B. , Kern, J. , Saenger, W. , Zouni, A. , & Biesiadka, J. (2005). Towards complete cofactor arrangement in the 3.0 angstrom resolution structure of Photosystem II. Nature, 438(7070), 1040–1044. 10.1038/nature04224 16355230

[gbi12322-bib-0087] Lynch, M. , & Conery, J. S. (2000). The evolutionary fate and consequences of duplicate genes. Science, 290(5494), 1151–1155. 10.1126/science.290.5494.1151 11073452

[gbi12322-bib-0088] Lyons, T. W. , Reinhard, C. T. , & Planavsky, N. J. (2014). The rise of oxygen in Earth's early ocean and atmosphere. Nature, 506(7488), 307–315. 10.1038/nature13068 24553238

[gbi12322-bib-0089] Magnabosco, C. , Moore, K. R. , Wolfe, J. M. , & Fournier, G. P. (2018). Dating phototropic microbial lineages with reticulate gene histories. Geobiology, 16, 179–189. 10.1111/gbi.12273 29384268 PMC5873394

[gbi12322-bib-0090] Marin, J. , Battistuzzi, F. U. , Brown, A. C. , & Hedges, S. B. (2017). The timetree of prokaryotes: New insights into their evolution and speciation. Molecular Biology and Evolution, 34, 437–446. 10.1093/molbev/msw245 27965376

[gbi12322-bib-0091] Matsunami, M. , Yoshioka, T. , Minoura, T. , Okano, Y. , & Muto, Y. (2011). Evolutionary features and intracellular behavior of the PRTB protein. Biochemical Genetics, 49(7–8), 458–473. 10.1007/s10528-011-9422-z 21274613

[gbi12322-bib-0092] Melo, T. B. , Frigaard, N. U. , Matsuura, K. , & Naqvi, K. R. (2000). Electronic energy transfer involving carotenoid pigments in chlorosomes of two green bacteria: *Chlorobium tepidum* and *Chloroflexus aurantiacus* . Spectrochimica Acta Part A: Molecular and Biomolecular Spectroscopy, 56(10), 2001–2010. 10.1016/S1386-1425(00)00289-4 10989892

[gbi12322-bib-0093] Mukhopadhyay, J. , Crowley, Q. G. , Ghosh, S. , Ghosh, G. , Chakrabarti, K. , Misra, B. , & Bose, S. (2014). Oxygenation of the Archean atmosphere: New paleosol constraints from eastern India. Geology, 42(10), 923–926. 10.1130/G36091.1

[gbi12322-bib-0094] Murray, J. W. (2012). Sequence variation at the oxygen‐evolving centre of Photosystem II: a new class of ‘rogue’ cyanobacterial D1 proteins. Photosynthesis Research, 110(3), 177–184. 10.1007/s11120-011-9714-5 22187288

[gbi12322-bib-0095] Nisbet, E. G. , & Fowler, C. F. R. (2014). The early history of life In McConnaghay K. D. M. & Schlesinger W. H. (Eds.), Treatise on geochemistry (2nd ed., Vol. 10, pp. 1–42). Amsterdam, the Netherlands: Elsevier Science.

[gbi12322-bib-0096] Nitschke, W. , & Rutherford, A. W. (1991). Photosynthetic reaction centers ‐ Variations on a common structural theme. Trends in Biochemical Sciences, 16(7), 241–245. 10.1016/0968-0004(91)90095-D 1926331

[gbi12322-bib-0097] Niwa, S. , Yu, L. J. , Takeda, K. , Hirano, Y. , Kawakami, T. , Wang‐Otomo, Z. Y. , & Miki, K. (2014). Structure of the LH1‐RC complex from *Thermochromatium tepidum* at 3.0 A. Nature, 508(7495), 228–232. 10.1038/nature13197 24670637

[gbi12322-bib-0098] Noguchi, T. , Mitsuka, T. , & Inoue, Y. (1994). Fourier transform infrared spectrum of the radical cation of beta‐carotene photoinduced in Photosystem II. FEBS Letters, 356(2–3), 179–182. 7805833 10.1016/0014-5793(94)01263-6

[gbi12322-bib-0099] Nurnberg, D. J. , Morton, J. , Santabarbara, S. , Telfer, A. , Joliot, P. , Antonaru, L. A. , & Rutherford, A. W. (2018). Photochemistry beyond the red limit in chlorophyll f‐containing photosystems. Science, 360(6394), 1210–1213. 10.1126/science.aar8313 29903971

[gbi12322-bib-0100] Nutman, A. P. , Bennett, V. C. , & Friend, C. R. L. (2017). Seeing through the magnetite: Reassessing Eoarchean atmosphere composition from Isua (Greenland) ≥3.7 Ga banded iron formations. Geoscience Frontiers, 8, 1233–1240. 10.1016/j.gsf.2017.02.008

[gbi12322-bib-0101] Orf, G. S. , Gisriel, C. , & Redding, K. E. (2018). Evolution of photosynthetic reaction centers: Insights from the structure of the heliobacterial reaction center. Photosynthesis Research, 138, 11–37. 10.1007/s11120-018-0503-2 29603081

[gbi12322-bib-0102] Planavsky, N. J. , Asael, D. , Hofmann, A. , Reinhard, C. T. , Lalonde, S. V. , Knudsen, A. , & Rouxel, O. J. (2014). Evidence for oxygenic photosynthesis half a billion years before the Great Oxidation Event. Nature Geoscience, 7(4), 283–286. 10.1038/ngeo2122

[gbi12322-bib-0103] Popelkova, L. , & Yocum, C. F. (2011). PsbO, the manganese‐stabilizing protein: Analysis of the structure‐function relations that provide insights into its role in Photosystem II. Journal of Photochemistry and Photobiology B‐Biology, 104(1–2), 179–190. 10.1016/j.jphotobiol.2011.01.015 21316983

[gbi12322-bib-0104] Qu, Y. G. , Zhu, S. X. , Whitehouse, M. , Engdahl, A. , & McLoughlin, N. (2018). Carbonaceous biosignatures of the earliest putative macroscopic multicellular eukaryotes from 1630 Ma Tuanshanzi Formation, north China. Precambrian Research, 304, 99–109. 10.1016/j.precamres.2017.11.004

[gbi12322-bib-0105] Riding, R. , Fralick, P. , & Liang, L. Y. (2014). Identification of an Archean marine oxygen oasis. Precambrian Research, 251, 232–237. 10.1016/j.precamres.2014.06.017

[gbi12322-bib-0106] Roose, J. L. , Frankel, L. K. , Mummadisetti, M. P. , & Bricker, T. M. (2016). The extrinsic proteins of Photosystem II: update. Planta, 243(4), 889–908. 10.1007/s00425-015-2462-6 26759350

[gbi12322-bib-0107] Rosing, M. T. (1999). C‐13‐depleted carbon microparticles in >3700‐Ma sea‐floor sedimentary rocks from west Greenland. Science, 283(5402), 674–676. 10.1126/science.283.5402.674 9924024

[gbi12322-bib-0108] Rosing, M. T. , & Frei, R. (2004). U‐rich Archaean sea‐floor sediments from Greenland ‐ indications of >3700 Ma oxygenic photosynthesis. Earth and Planetary Science Letters, 217(3–4), 237–244. 10.1016/S0012-821x(03)00609-5

[gbi12322-bib-0109] Ruffle, S. V. , Wang, J. , Johnston, H. G. , Gustafson, T. L. , Hutchison, R. S. , Minagawa, J. , … Sayre, R. T. (2001). Photosystem II peripheral accessory chlorophyll mutants in Chlamydomonas reinhardtii. Biochemical characterization and sensitivity to photo‐inhibition. Plant Physiology, 127(2), 633–644. 11598237 PMC125098

[gbi12322-bib-0110] Rutherford, A. W. (1989). Photosystem II, the water‐splitting enzyme. Trends in Biochemical Sciences, 14(6), 227–232. 10.1016/0968-0004(89)90032-7 2669240

[gbi12322-bib-0111] Rutherford, A. W. , & Faller, P. (2003). Photosystem II: Evolutionary perspectives. Philosophical Transactions of the Royal Society B‐Biological Sciences, 358(1429), 245–253. 10.1098/rstb.2002.1186 PMC1693113 12594932

[gbi12322-bib-0112] Rutherford, A. W. , & Nitschke, W. (1996). Photosystem II and the quinone–iron‐containing reaction centers In Baltscheffsky H. (Ed.), Origin and evolution of biological energy conversion (pp. 143–175). New York, NY: VCH.

[gbi12322-bib-0113] Rutherford, A. W. , Osyczka, A. , & Rappaport, F. (2012). Back‐reactions, short‐circuits, leaks and other energy wasteful reactions in biological electron transfer: Redox tuning to survive life in O_2_ . FEBS Letters, 586(5), 603–616. 10.1016/j.febslet.2011.12.039 22251618

[gbi12322-bib-0114] Rutherford, A. W. , Paterson, D. R. , & Mullet, J. E. (1981). A light‐induced spin‐polarized triplet detected by EPR in Photosystem II reaction centers. Biochimica et Biophysica Acta (BBA)‐Bioenergetics, 635(2), 205–214. 6263330 10.1016/0005-2728(81)90020-7

[gbi12322-bib-0115] Sadekar, S. , Raymond, J. , & Blankenship, R. E. (2006). Conservation of distantly related membrane proteins: Photosynthetic reaction centers share a common structural core. Molecular Biology and Evolution, 23(11), 2001–2007. 10.1093/molbev/msl079 16887904

[gbi12322-bib-0116] Sallstedt, T. , Bengtson, S. , Broman, C. , Crill, P. M. , & Canfield, D. E. (2018). Evidence of oxygenic phototrophy in ancient phosphatic stromatolites from the Paleoproterozoic Vindhyan and Aravalli Supergroups, India. Geobiology, 16, 139–159. 10.1111/gbi.12274 29380943

[gbi12322-bib-0117] Sanchez‐Baracaldo, P. (2015). Origin of marine planktonic cyanobacteria. Scientific Reports, 5, 17418 10.1038/srep17418 26621203 PMC4665016

[gbi12322-bib-0118] Sanchez‐Baracaldo, P. , Raven, J. A. , Pisani, D. , & Knoll, A. H. (2017). Early photosynthetic eukaryotes inhabited low‐salinity habitats. Proceedings of the National Academy of Sciences of USA, 114, E7737–E7745. 10.1073/pnas.1620089114 PMC5603991 28808007

[gbi12322-bib-0119] Santabarbara, S. , Bordignon, E. , Jennings, R. C. , & Carbonera, D. (2002). Chlorophyll triplet states associated with Photosystem II of thylakoids. Biochemistry, 41(25), 8184–8194. 12069611 10.1021/bi0201163

[gbi12322-bib-0120] Satkoski, A. M. , Beukes, N. J. , Li, W. , Beard, B. L. , & Johnson, C. M. (2015). A redox‐stratified ocean 3.2 billion years ago. Earth and Planetary Science Letters, 430, 43–53.

[gbi12322-bib-0121] Schidlowski, M. (1988). A 3,800‐million‐year isotopic record of life from carbon in sedimentary‐rocks. Nature, 333(6171), 313–318. 10.1038/333313a0

[gbi12322-bib-0122] Schirrmeister, B. E. , de Vos, J. M. , Antonelli, A. , & Bagheri, H. C. (2013). Evolution of multicellularity coincided with increased diversification of cyanobacteria and the Great Oxidation Event. Proceedings of the National Academy of Sciences of USA, 110(5), 1791–1796. 10.1073/pnas.1209927110 PMC3562814 23319632

[gbi12322-bib-0123] Schirrmeister, B. E. , Gugger, M. , & Donoghue, P. C. J. (2015). Cyanobacteria and the great oxidation event: Evidence from genes and fossils. Palaeontology, 58(5), 769–785. 10.1111/pala.12178 26924853 PMC4755140

[gbi12322-bib-0124] Schirrmeister, B. E. , Sanchez‐Baracaldo, P. , & Wacey, D. (2016). Cyanobacterial evolution during the Precambrian. International Journal of Astrobiology, 15, 187–204. 10.1017/s1473550415000579

[gbi12322-bib-0125] Schopf, J. W. , Kitajima, K. , Spicuzza, M. J. , Kudryavtsev, A. B. , & Valley, J. W. (2018). SIMS analyses of the oldest known assemblage of microfossils document their taxon‐correlated carbon isotope compositions. Proceedings of the National Academy of Sciences of USA, 115(1), 53–58. 10.1073/pnas.1718063115 PMC5776830 29255053

[gbi12322-bib-0126] Sergeev, V. N. , Gerasimenko, L. M. , & Zavarzin, G. A. (2002). Proterozoic history and present state of cyanobacteria. Mikrobiologiia, 71(6), 725–740. 12526193

[gbi12322-bib-0127] Shao, S. , Cardona, T. , & Nixon, P. J. (2018). Early emergence of the FtsH proteases involved in Photosystem II repair. Photosynthetica, 56, 163–177. 10.1007/s11099-018-0769-9

[gbi12322-bib-0128] Sharma, P. P. , & Wheeler, W. C. (2014). Cross‐bracing uncalibrated nodes in molecular dating improves congruence of fossil and molecular age estimates. Frontiers in Zoology, 11 10.1186/s12983-014-0057-x

[gbi12322-bib-0129] Sheldon, N. D. (2006). Precambrian paleosols and atmospheric CO_2_ levels. Precambrian Research, 147(1–2), 148–155. 10.1016/j.precamres.2006.02.004

[gbi12322-bib-0130] Shi, T. , Bibby, T. S. , Jiang, L. , Irwin, A. J. , & Falkowski, P. G. (2005). Protein interactions limit the rate of evolution of photosynthetic genes in cyanobacteria. Molecular Biology and Evolution, 22(11), 2179–2189. 10.1093/molbev/msi216 16014867

[gbi12322-bib-0131] Shih, P. M. , Hemp, J. , Ward, L. M. , Matzke, N. J. , & Fischer, W. W. (2017). Crown group Oxyphotobacteria postdate the rise of oxygen. Geobiology, 15(1), 19–29. 10.1111/gbi.12200 27392323

[gbi12322-bib-0132] Shih, P. M. , & Matzke, N. J. (2013). Primary endosymbiosis events date to the later Proterozoic with cross‐calibrated phylogenetic dating of duplicated ATPase proteins. Proceedings of the National Academy of Sciences of USA, 110(30), 12355–12360. 10.1073/pnas.1305813110 PMC3725117 23776247

[gbi12322-bib-0133] Shih, P. M. , Ward, L. M. , & Fischer, W. W. (2017). Evolution of the 3‐hydroxypropionate bicycle and recent transfer of anoxygenic photosynthesis into the Chloroflexi. Proceedings of the National Academy of Sciences of USA, 114(40), 10749–10754. 10.1073/pnas.1710798114 PMC5635909 28923961

[gbi12322-bib-0134] Sievers, F. , Wilm, A. , Dineen, D. , Gibson, T. J. , Karplus, K. , Li, W. Z. , … Higgins, D. G. (2011). Fast, scalable generation of high‐quality protein multiple sequence alignments using Clustal Omega. Molecular Systems Biology, 7 10.1038/msb.2011.75 PMC3261699 21988835

[gbi12322-bib-0135] Sims, P. A. , Mann, D. G. , & Medlin, L. K. (2006). Evolution of the diatoms: Insights from fossil, biological and molecular data. Phycologia, 45(4), 361–402. 10.2216/05-22.1

[gbi12322-bib-0136] Smit, M. A. , & Mezger, K. (2017). Earth's early O_2_ cycle suppressed by primitive continents. Nature Geoscience, 10(10), 788–792. 10.1038/Ngeo3030

[gbi12322-bib-0137] Soo, R. M. , Hemp, J. , Parks, D. H. , Fischer, W. W. , & Hugenholtz, P. (2017). On the origins of oxygenic photosynthesis and aerobic respiration in Cyanobacteria. Science, 355(6332), 1436–1440. 10.1126/science.aal3794 28360330

[gbi12322-bib-0138] Stamatakis, K. , Tsimilli‐Michael, M. , & Papageorgiou, G. C. (2014). On the question of the light‐harvesting role of beta‐carotene in Photosystem II and Photosystem I core complexes. Plant Physiology and Biochemistry, 81, 121–127. 10.1016/j.plaphy.2014.01.014 24529497

[gbi12322-bib-0139] Sugiura, M. , Nakamura, M. , Koyama, K. , & Boussac, A. (2015). Assembly of oxygen‐evolving Photosystem II efficiently occurs with the apo‐Cytb(559) but the holo‐Cytb(559) accelerates the recovery of a functional enzyme upon photoinhibition. Biochimica et Biophysica Acta (BBA)‐Bioenergetics, 1847(2), 276–285. 10.1016/j.bbabio.2014.11.009 25481108

[gbi12322-bib-0140] Svensson, B. , Vass, I. , & Styring, S. (1991). Sequence‐Analysis of the D1 and D2 Reaction Center Proteins of Photosystem‐Ii. Zeitschrift Fur Naturforschung Section C‐A Journal of Biosciences, 46(9–10), 765–776. 10.1515/znc-1991-9-1008 1750944

[gbi12322-bib-0141] Tachibana, Y. , Vayssieres, L. , & Durrant, J. R. (2012). Artificial photosynthesis for solar water‐splitting. Nature Photonics, 6(8), 511–518. 10.1038/nphoton.2012.175

[gbi12322-bib-0142] Telfer, A. (2002). What is beta‐carotene doing in the Photosystem II reaction centre? Philosophical Transactions of the Royal Society of London Series B‐Biological Sciences, 357(1426), 1431–1439. 10.1098/rstb.2002.1139 PMC1693050 12437882

[gbi12322-bib-0143] Telfer, A. , Dhami, S. , Bishop, S. M. , Phillips, D. , & Barber, J. (1994). Beta‐carotene quenches singlet oxygen formed by isolated Photosystem II reaction centers. Biochemistry, 33(48), 14469–14474. 10.1021/bi00252a013 7981207

[gbi12322-bib-0144] Tice, M. M. , & Lowe, D. R. (2004). Photosynthetic microbial mats in the 3,416‐Myr‐old ocean. Nature, 431(7008), 549–552. 10.1038/nature02888 15457255

[gbi12322-bib-0145] Tomitani, A. , Knoll, A. H. , Cavanaugh, C. M. , & Ohno, T. (2006). The evolutionary diversification of cyanobacteria: Molecular‐phylogenetic and paleontological perspectives. Proceedings of the National Academy of Sciences of USA, 103(14), 5442–5447. 10.1073/pnas.0600999103 PMC1459374 16569695

[gbi12322-bib-0146] Tsukatani, Y. , Romberger, S. P. , Golbeck, J. H. , & Bryant, D. A. (2012). Isolation and characterization of homodimeric Type‐I reaction center complex from *Candidatus* Chloracidobacterium thermophilum, an aerobic chlorophototroph. Journal of Biological Chemistry, 287(8), 5720–5732. 10.1074/jbc.M111.323329 22184116 PMC3285344

[gbi12322-bib-0147] Umena, Y. , Kawakami, K. , Shen, J. R. , & Kamiya, N. (2011). Crystal structure of oxygen‐evolving Photosystem II at a resolution of 1.9 A. Nature, 473(7345), 55–60. 10.1038/nature09913 21499260

[gbi12322-bib-0148] Vass, I. , & Cser, K. (2009). Janus‐faced charge recombinations in Photosystem II photoinhibition. Trends in Plant Science, 14(4), 200–205. 10.1016/j.tplants.2009.01.009 19303349

[gbi12322-bib-0149] Wang, X. L. , Planavsky, N. J. , Hofmann, A. , Saupe, E. E. , De Corte, B. P. , Philippot, P. , & Konhauser, K. O. (2018). A Mesoarchean shift in uranium isotope systematics. Geochimica Et Cosmochimica Acta, 238, 438–452. 10.1016/j.gca.2018.07.024

[gbi12322-bib-0150] Ward, L. M. , Kirschvink, J. L. , & Fischer, W. W. (2016). Timescales of oxygenation following the evolution of oxygenic photosynthesis. Origins of Life and Evolution of Biospheres, 46(1), 51–65. 10.1007/s11084-015-9460-3 26286084

[gbi12322-bib-0151] Wegener, K. M. , Nagarajan, A. , & Pakrasi, H. B. (2015). An atypical psbA gene encodes a sentinel D1 protein to form a physiologically relevant inactive Photosystem II complex in cyanobacteria. Journal of Biological Chemistry, 290(6), 3764–3774. 10.1074/jbc.M114.604124 25525275 PMC4319040

[gbi12322-bib-0152] Xiao, S. , Knoll, A. H. , Yuan, X. , & Pueschel, C. M. (2004). Phosphatized multicellular algae in the Neoproterozoic Doushantuo Formation, China, and the early evolution of florideophyte red algae. American Journal of Botany, 91(2), 214–227. 10.3732/ajb.91.2.214 21653378

[gbi12322-bib-0153] Yang, E. C. , Boo, S. M. , Bhattacharya, D. , Saunders, G. W. , Knoll, A. H. , Fredericq, S. , & Yoon, H. S. (2016). Divergence time estimates and the evolution of major lineages in the florideophyte red algae. Scientific Reports, 6, 21361 10.1038/srep21361 26892537 PMC4759575

[gbi12322-bib-0154] Yang, Z. , & Rannala, B. (2006). Bayesian estimation of species divergence times under a molecular clock using multiple fossil calibrations with soft bounds. Molecular Biology and Evolution, 23(1), 212–226. 10.1093/molbev/msj024 16177230

[gbi12322-bib-0155] Yao, D. C. , Brune, D. C. , & Vermaas, W. F. (2012). Lifetimes of Photosystem I and II proteins in the cyanobacterium *Synechocystis* sp. PCC 6803. FEBS Letters, 586(2), 169–173. 10.1016/j.febslet.2011.12.010 22197103

[gbi12322-bib-0156] Zeng, Y. H. , Feng, F. Y. , Medova, H. , Dean, J. , & Koblizek, M. (2014). Functional Type 2 photosynthetic reaction centers found in the rare bacterial phylum Gemmatimonadetes. Proceedings of the National Academy of Sciences of USA, 111(21), 7795–7800. 10.1073/pnas.1400295111 PMC4040607 24821787

