# Supplementary material for: Early Archean origin of Photosystem II
Source: Geobiology. 2018 Nov 9;17(2):127–50. doi: 10.1111/gbi.12322 (PMC6492235; doi:10.1111/gbi.12322)
Supplement: Supplementary file 1 [file GBI-17-127-s001.docx]

**Contents**

*Supplementary discussion, page 1*

*Supplementary tables, page 9*

*Supplementary figures, page 11*

*References, page 14*

**Supplementary discussion**

*Diversification of phototrophic lineages*

Cardona (2015) suggested based on the phylogeny of reaction center proteins that some of the earliest events in the evolution of photosynthesis, like the divergence of Type I and Type II reaction centers, predated the diversification event that gave rise to the known diversity of phototrophs. In accord with this, several recent studies have highlighted that most, if not all, the described groups of phototrophs appear to be much younger than the origin of photosynthesis itself, with every group having close non-photosynthetic relatives. This was first pointed out by Cardona (2015), but see also Fischer et al. (2016). For example, Shih, Hemp, et al. (2017) reported a molecular clock of Cyanobacteria and their closest non-photosynthetic relatives the Sericytochromatia and the Melainabacteria (Soo et al., 2014; Soo et al., 2017), in which the divergence of *Gloeobacter* was estimated to postdate the GOE. Such a late date contrasts with other molecular clocks that placed the same node before the GOE, in the range of 2.8 to 2.6 Ga (Sanchez-Baracaldo, 2015). Along the same lines, Magnabosco et al. (2018) estimated that the divergence of *Gloeobacter* likely occurred around the GOE, between 2.7 to 2.2 Ga depending on the calibration choice and evolutionary model. In the case of phototrophic Chloroflexi, Shih, Ward, et al. (2017) estimated an age of 1.0 Ga for this group and Magnabosco et al. (2018) between 2.1 and 1.0 Ga, depending on the models used. The age estimates derived from the evolution of Type II reaction centers presented in this work are consistent with the molecular clock studies mentioned above, as discussed in the main text.

In the same work, Magnabosco et al. (2018) also timed the origin of phototrophic Chlorobi, having homodimeric Type I reaction centers, at about 1.7 Ga. This date is consistent with biomarkers specific to phototrophic Chlorobi showing up in the rock record for the first time about 1.6 Ga ago (Brocks et al., 2005). In agreement with this, a molecular clock analysis of Type I reaction center proteins also timed the MRCA of phototrophic Chlorobi in the range of 2.1 to 1.6 Ga (Cardona, 2018).

It is worth noting, however, that even permitting for high levels of uncertainty in the different molecular clock studies listed above, it looks like the most recent common ancestors of each of these phototrophic groups, anoxygenic or oxygenic, occurred long after the origin of photosynthesis. Indeed, a molecular clock of nitrogenases and their related enzymes of the chlorophyll synthesis pathway by Boyd et al. (2011) showed that the duplication event leading to protocholorphyllide and chlorophyllide reductases was likely to have occurred about a billion years before the diversification of the known groups of phototrophs. The data presented in this work adds an extra dimension to the timing of the emergence of the known described groups of phototrophs by following the history of the reaction centers themselves as direct proxies for the evolution of photosynthesis. We can conclude then that when each of the known groups of phototrophs diversified, and this includes Cyanobacteria as well, their reaction centers had already reached a high-degree of specialization regardless of any gains or losses of phototrophy along this evolutionary trajectory.

From this perspective, the early duplication of **D0** implies that the Sericytochromatia and Melainbacteria classes of non-photosynthetic Cyanobacteria originated after losses of photosynthesis, as they specialized in heterotrophic lifestyles (e.g. symbionts in the gut, subsurface) and were supported by the primary productivity of their photosynthetic cousins. The loss of oxygenic photosynthesis is a relatively common process across the tree of life, several well-known examples are: the secondary loss of all PSII genes by the symbiont *Atelocyanobacterium talassa* (Thompson et al., 2012; Cornejo-Castillo et al., 2016), the complete loss of photosynthesis of the cyanobacterium endosymbiont of *Epithemia turgida* (Nakayama et al., 2014), the case of parasitic apicomplexa (Moore et al., 2008) and holoparasitic angiosperms (Ravin et al., 2016).

*Rates of evolution*

The phenomenon of an initial fast rate of evolution followed by an exponential decrease demands an explanation. A mechanism that can lead to accelerated rates of evolution is gene duplication followed by functional innovation (Lynch & Conery, 2000; Innan & Kondrashov, 2010). In such scenario the fast rates of evolution at the nodes representing **K** and **D0** may be linked to each respective duplication and the initiation of heterodimerization. Studies on the rates of evolution of duplicated genes suggest that immediately after a duplication there is an acceleration of the rate of the gene that acquires a new function, followed by a slow-down of the rate (Duda & Palumbi, 1999; Lynch & Conery, 2000; Rosello & Kondrashov, 2014). It is thought that the acceleration of the rate is due to the relaxation of purifying selection (Jordan et al., 2004). However, the timing of these events seem to be completed within tens of millions of years (Rosello & Kondrashov, 2014) and not in the scale of a billion years as detected for the evolution of the core Type II reaction center proteins (Fig. 5A). In addition, duplication of PSI and PSII genes in Cyanobacteria are common across all taxonomic levels (Cardona, 2015; Cardona et al., 2015), but in none of the cases the predicted relaxation of purifying selection has led to accelerations in the rates matching those of **K** and **D0**, in such a way that it would lead to a drastic and rapid decrease in the levels of sequence identity between paralogs. Consistent with this, it has been shown that even duplicated genes evolve slower than singletons despite the initial acceleration of the rate (Jordan et al., 2004). Nevertheless, it is plausible that major evolutionary innovations such as the origin of photosynthesis or aerobic respiration may have had a more pronounced and prolonged effect in the rate of evolution of duplicated genes essential for these processes.

Another possibility is the temperature dependent deamination of cytosine, as suggested by Lewis and coworkers (2016). They calculated that as the Earth cooled during its first 4 Ga, the rate of spontaneous mutation would have fallen exponentially by a factor of more than 4000. That is to say, the rate of spontaneous mutation during the earliest stages in the history of life would have been about three orders of magnitude greater than those observed since the Proterozoic. Lewis and coworkers (2016) calculated that 50% of all spontaneous mutations occurred in the first 0.2 Ga, which matches well with the exponential decay trend seen in Fig. 5A, especially if the origin of photosynthesis occurred before 3.8 Ga. Two possible limitations to this scenario are noted: 1) no hyperthermophilic phototrophs are known, and 2) currently there is no evidence that organisms in thermal environments display faster rates of evolution (Groussin & Gouy, 2011).

A third possibility is higher UV radiation on the planet’s surface during the early Earth in the absence of an ozone layer, which could have resulted in rates of DNA damage up to three orders of magnitude greater than in present day Earth, as calculated by Cockell (2000); this higher rate of damage may have led as well to faster rates of change.

*Peculiar oxygen anomalies*

The following compilation is not meant to be exhaustive.

A long-standing debate on the early evolution of life is whether aerobic respiration predates oxygenic photosynthesis. This debate started with the realization that the core proteins of the cytochrome *c* oxidases, which catalyzes the reduction of oxygen to water, are almost universally conserved and appear to be much older than Cyanobacteria, see A. L. Ducluzeau et al. (2014) for a critical review. One example of this can be found in Brochier-Armanet et al. (2009) who wrote: “*A-O_2_Red are the most ancient O_2_Red and were already present prior to the divergence of major present-day bacterial and archaeal phyla, thus before the emergence of Cyanobacteria and oxygenic photosynthesis.*” Many ways to rationalize this anomaly have been put forward, including the possibility that O_2_ reductases originated to use the traces of oxygen (few nanomolar or less) that are expected in a world without oxygenic photosynthesis (Stolper et al., 2010) or that they originated from NO reductases (A. L. Ducluzeau et al., 2014).

Weiss et al. (2016) reported an automated minimal reconstruction of the proteome of the last universal common ancestor (LUCA). They suggested that this was an anaerobe, methanogenic and capable of nitrogen fixation. However, out of 355 predicted proteins 8 turned out to use oxygen or to deal with reactive oxygen species (ROS): more than those categorized as “nitrogen metabolism” (seven proteins) and “energy metabolism” (two proteins). Nevertheless, Weiss et al. (2016) dismissed these as artefacts.

These 8 proteins are the following:

1. Heme/copper-type cytochrome/quinol oxidase
2. Cu/Zn superoxide dismutase
3. Peroxiredoxin
4. Rubrerythrin
5. Homogentisate 1,2-dioxygenase
6. Aromatic ring-opening dioxygenase
7. Aromatic ring hydroxylase
8. Betacarotene, NADH:oxygen 3-oxidoreductase

The first one is the core subunit of O_2_ reductases, as seen above. The next three enzymes handle ROS. The great antiquity of ROS-handling enzymes had been noted before (Zamocky et al., 2000; Ouzounis et al., 2006; Knoops et al., 2007; Slesak et al., 2012). The next three enzymes have a role in the catabolism and oxidation of aromatic rings of tyrosine, phenylalanine and other aromatic and phenolic compounds. The last enzyme is a monooxygenase involved in the hydroxylation of the aromatic rings of carotenoids.

The evolution of cytochrome *bc* and *b*_6_*f* complexes have also hinted to the possibility of oxygenic photosynthesis appearing at a very early stage. Nitschke et al. (2010) wrote the following: “*Our presently most favoured rationalisation for the presence of heme c_i_ postulates that this cofactor allows a substantial reduction in lifetime of the semiquinone species during Q_i_ site turnover in the b_6_f complex, thus adapting the enzyme to an oxygenic environment.*

*Reasoning along these lines, we cannot help noticing that this hypothesis would impose a mind-boggling evolutionary scenario for the emergence of oxygenic photosynthesis: If heme c_i_ indeed evolved as a response to the presence of oxygen, we have to conclude that photosynthetic O_2_ production had already evolved prior to the node leading to Firmicutes and Cyanobacteria*.”

An examination of the phylogeny of oxygen-tolerant hydrogenases led to the following statement by Pandelia et al. (2012): “*The tree topologies shown in Figs. 3 and 4 suggest a provocative conclusion: 6C-hydrogenases* [oxygen-tolerant] *appear to have emerged prior to the divergence of Chlorobia/Heliobacteria on one and α-, β- and γ-proteobacterial radiations on the other side. In the traditional thinking that Heliobacteria and Firmicutes have diverged prior to the origin of oxygenic photosynthesis, the presence of 6C-enzymes in these species would indicate that their appearance pre-dates the emergence of light-driven O_2_ production. Therefore, either the 6C cluster appeared without an apparent link to O_2_-tolerance or we need to rethink our evolutionary history of oxygenic photosynthesis.*”

A recent paper by Kacar et al. (2017) indicated that the divergence of methanogenic archaeal Group 3 rubisco from photosynthetic bacterial Group 1 rubisco occurred as the latter acquired structural traits to deal with oxygen and to optimize oxygen-mediated regulation, but see also Nisbet et al. (2007). The phylogeny of these enzymes showed a deep divergence for Group 3 and Group 1 rubisco, which is expected for an archaeal/bacterial split. Rubisco is undoubtedly a very ancient enzyme probably as old as 3.5 Ga (Nisbet et al., 2007; Schopf et al., 2018). In agreement with this, Weiss et al. (2016) reported that rubisco would trace to LUCA if a single ancient event of HGT is allowed. Thus, it is not surprising that this enzyme displays very slow rates of evolution. In fact, between all Group 1 rubisco, which include all cyanobacterial and proteobacterial rubisco, the level of sequence identity is not lower than 78% (Kacar et al., 2017). In contrast, the level of sequence identity between archaeal Group 3 rubisco and bacterial Group 1 rubisco is 39%. At these slow rates, even slower than the rates of evolution of D1 and D2, the divergence of Group 1 and Group 3 rubisco would be placed in the early Archean.

Gold et al. (2017) applied a molecular clock approach to time the evolution of sterol biosynthesis proteins, the product of ancient duplications. They studied squalene monooxygenase and oxidosqualene cyclase, both of which use oxygen. They noted that the ancestral node to these oxygen-using enzymes was timed around the GOE: however, the duplication event leading to the acquisition of oxygen-using capabilities was deep in the early Archean.

A.-L. Ducluzeau and Nitschke (2016) have also pointed out the deep dichotomies that exists in several metabolic processes, which arguably may have occurred for the integration of oxygen-using enzymes. The first example is the dichotomy of the heme synthesis pathway: one goes via protoporphyrin IX and can incorporate several oxygen-using enzymes, and a second via sirohydrochlorin found in many anaerobes. A similar dichotomy exists in the biosynthesis pathway of menaquinone, the men pathway and the futalosine pathway; in the pathway for the assembly of iron-sulfur clusters, the Isc and Suf systems; and in the aerobic and anaerobic pathways for the synthesis of cobalamin. A.-L. Ducluzeau and Nitschke (2016) highlighted that oxygen-using enzymes are asymmetrically distributed in these pathways. It should be noted that the phylogenetic relationship of many enzymes in these metabolic processes remain to be elucidated, yet the distribution of these dichotomic pathways across prokaryotes argue against very recent divergences (e.g. post-GOE).

For example, the aerobic pathway for the synthesis of cobalamin uses an enzyme homologous to Mg-chelatase of the chlorophyll synthesis pathway to insert Co into the precursor ring. In the aerobic pathway this is done towards the end of the pathway *after* the oxygen-requiring step, while in the anaerobic pathway Co insertion occurs at the beginning of the pathway by a different chelatase unrelated to Mg-chelatase, see Sousa et al. (2013) and reference therein. If Mg-chelatase, at the origin of photosynthesis, emerged from Co-chelatase: would not that imply that the aerobic pathway for the synthesis of cobalamin predated the origin of photosynthesis? On the other hand, if Co-chelatase originated from Mg-chelatase then it could be postulated that the aerobic cobalamin synthesis pathway emerged after the evolution of oxygenic photosynthesis. However, all Mg-chelatase subunits used in photosynthesis are monophyletic to the exclusion of Co-chelatase’s paralogs (Sousa et al., 2013). The oxygen-dependent pathway for the synthesis of cobalamin is catalyzed by two enzymes, CobG or CobZ, both of which use oxygen (Heldt et al., 2005). One could postulate therefore that the aerobic pathway was at first an entirely anaerobic path and that the oxygen-dependent step was catalyzed by an alternative enzyme that did not require oxygen, yet an oxygen-independent enzyme catalyzing this step, to the best of our knowledge, has not been reported. This conundrum is easily resolved if primordial forms of oxygenic photosynthesis appeared early in the evolutionary history of life.

David and Alm (2011) using a combined phylogenomic/molecular clock approach showed that a major diversification event took place in prokaryotes starting about 3.4-3.5 Ga and peaking about 3.2 Ga. The authors claimed that in this period of evolutionary innovation 27% of major modern gene families appeared. The authors stated: *“Our chronologies of oxygen and redox-sensitive metal and compound utilization suggest ancient increases in oxygen bioavailability, as well as an Archaean biosphere with some of the basic genetic components required for oxygenic photosynthesis and respiration”*, and yet their molecular clock (shown in their Supplementary Figure 13) timed the MRCA of Cyanobacteria at about 2.0 Ga, and the divergence of the Cyanobacteria and Chloroflexi phyla at about 3.0 Ga.

More recently, Granold et al. (2018) reported that the last evolving amino acids were only incorporated into the universal genetic code because of oxygen. The authors stated “*Our data indicate that in demanding building blocks with more versatile redox chemistry, biospheric molecular oxygen triggered the selective fixation of the last amino acids in the genetic code.*” But how could the universal genetic code have been only “fixed” in the late Archean or around the GOE, when the divergence of Bacteria and Archaea may have occurred more than 3.5 Ga ago (Marin et al., 2017; Schopf et al., 2018)?

We do **not** claim that oxygenic photosynthesis originated in the LUCA. Nonetheless, all of the above apparent anomalies could be conveniently explained if primordial forms of photosynthetic water oxidation appeared at an early stage in the evolutionary history of Bacteria and photosynthesis. In consequence, limited amounts of bioavailable oxygen would affect metabolic processes and allow the emergence of aerobic respiration and other oxygen-using and ROS-handling enzymes from an early time and long before the most recent common ancestor of described Cyanobacteria, even if other complex geological processes delayed the oxygenation of the atmosphere until the GOE (Smit & Mezger, 2017; Bindeman et al., 2018).

**Supplementary tables and figures**

**Table S1.** Percentage of sequence identity as a function of divergence time

| **Pairs of relationships** | | **Divergence time**^a^ **(Ma)** | **Sequence Identity (%)** | |
| --- | --- | --- | --- | --- |
|  |  |  | **D1 v D1** | **D2 v D2** |
| 1 | *Arabidopsis* *thaliana* vs *Populus trichocarpa* | 104.6 | 99.71 | 99.15 |
| 2 | *Chloranthus* *spicatus* vs *Liriodendron tulipifera* | 173.2 | 99.15 | 99.43 |
| 3 | *Arabidopsis* vs *Sorghum bicolor* | 186.2 | 98.58 | 97.73 |
| 4 | *Arabidopsis* vs *Chloranthus spicatus* | 186.2 | 98.86 | 98.01 |
| 5 | *Arabidopsis* vs *Illicium oligandrum* | 186.2 | 98.86 | 98.30 |
| 6 | *Arabidopsis* vs *Nymphaea alba* | 186.2 | 98.86 | 98.58 |
| 7 | *Arabidopsis* vs *Amborella trichopoda* | 186.2 | 99.15 | 98.01 |
| 8 | *Welwitschia mirabilis* vs *Pinus thunbergii* | 215.65 | 96.31 | 97.45 |
| 9 | *Welwitschia* vs *Cryptomeria japonica* | 228.25 | 98.01 | 97.45 |
| 10 | *Welwitschia* vs *Ginkgo bilboa* | 236.75 | 97.16 | 97.16 |
| 11 | *Welwitschia* vs *Cycas talitungensis* | 336.5 | 92.35 | 97.45 |
| 12 | *Arabidopsis* vs *Psilotum nudum* | 421.1 | 97.73 | 96.03 |
| 13 | *Arabidopsis* vs *Selaginella moellendorfii* | 435 | 96.88 | 93.76 |
| 14 | *Arabidopsis* vs *Anthoceros formosae* | 731.2 | 97.45 | 96.31 |
| 15 | *Arabidopsis* vs *Physcomitrella patens* | 731.2 | 97.16 | 96.60 |
| 16 | *Arabidopsis* vs *Marchantia polymorpha* | 745.5 | 96.60 | 96.60 |
| 17 | *Arabidopsis* vs *Chondrus crispus* | 943 | 89.16 | 87.81 |
| 18 | *Arabidopsis* vs *Calliarthron tuberculosum* | 943 | 89.44 | 86.685 |
| 19 | *Arabidopsis* vs *Pyropia haitanensis* | 1060 | 88.33 | 86.968 |
| 20 | *Arabidopsis* vs *Porphyra purpurea* | 1060 | 88.05 | 86.968 |
| 21 | *Arabidopsis* vs *Porphyridium purpureum* | 1194 | 90.00 | 86.685 |
| 22 | *Arabidopsis* vs *Cyanidioschyzon merolae* | 1500 | 87.70 | 85.83 |
| 23 | *Arabidopsis* vs *Galdieria sulphuraria* | 1500 | 86.94 | 86.11 |
| *Cyanidioschyzon* vs *Gloeobacter violaceus*^b^ | |  | 81.66 | 80.73 |
| *Cyanidioschyzon* vs *Gloeobacter violaceus* | |  | 78.05 |  |
| *Cyanidioschyzon* vs *Gloeobacter violaceus* | |  | 77.90 |  |
| *Arabidopsis* vs *Gloeobacter violaceus* | |  | 81.66 | 77.68 |
| *Arabidopsis* vs *Gloeobacter violaceus* | |  | 77.50 |  |
| *Arabidopsis* vs *Gloeobacter violaceus* | |  | 76.66 |  |

| **Pairs of relationships** | **RC proteins** | **Sequence Identity (%)** |
| --- | --- | --- |
| *Cyanidioschyzon merolae* | D2 v D1 | 29.72 |
| *Thermosynechococcus elongatus* | D2 v D1 | 27.77 |
| *Nostoc* sp. PCC 7120 | D2 v D1 | 30.83 |
| *Nostoc* sp. PCC 7120 vs *Chloroflexus* *aurantiacus* | D1 v L | 16.94 |
| *Nostoc* sp. PCC 7120 vs *Oscillochloris* *trichoides* | D1 v L | 15.83 |
| *Nostoc* sp. PCC 7120 vs *Erythrobacter* *citreus* | D1 v L | 19.16 |
| *Nostoc* sp. PCC 7120 vs Uncult. proteobacterium | D1 v L | 14.16 |
| *Thermosynechococcus* *elongatus* | D2 v PsaB | ≤ 5^c^ |

^a^The first 16 values are taken as the average of the hard minimum and soft maximum ages suggested by Clarke et al. (2011) and the last 7 values were taken from Yang et al. (2016)

^b^The genome of *Gloeobacter violaceous* has five *psbA* genes encoding three distinct dominant forms of D1 and a single *psbD* gene encoding a D2 protein.

^c^Determined by structural overlap of the reaction center proteins (Cardona, 2015).

**Table S2.** Fit of a linear regression of data in Fig. 2

| **Curve fit** (*y = a + b · x*) |  |
| --- | --- |
| Adj. R^2^ | 0.75833 |
| Slope (*b*) | -0.00889 ± 0.00114 |
| Intercept (*a*) | 100.03502 ± 0.8961 |

**Table S3.** Fit of a single component exponential decay function of data in Fig. 5A

| **Curve fit** (*y = A1· e^-x/t1^ + y0*) | **D1/D2** | **L//M** |
| --- | --- | --- |
| Adj. R^2^ | 0.73777 | 0.67032 |
| y0 | 4.19933 x 10^-5^ ± 1.06279 x 10^-5^ | 1.41889 x 10^-4^ ± 6.00278 x 10^-5^ |
| A1 | 3.62152 x 10^-7^ ± 2.99726 x 10^-7^ | 3.8073 x 10^-8^ ± 7.78163 x 10^-8^ |
| t1 | -349.97659 ± 40.20203 | -240.11954 ± 46.28278 |

**Table S4.** Fit of power law function of data in Fig. 5C

| **Curve fit** (*y = a · x^b^*) |  |
| --- | --- |
| Adj. R^2^: | 0.98114 |
| *a* | 12435.67171 ± 2465.59376 |
| *b* | -0.8299 ± 0.03242 |

|  | **Calibration 1**  **3.5 ± 0.05 (Ga)** | **Calibration 1**  **3.8 ± 0.05** | **Calibration 2**  **3.5 ± 0.05** | **Calibration 2**  **3.8 ± 0.05** |
| --- | --- | --- | --- | --- |
| Adj. R^2^ | 0.99882 | 0.99957 | 0.99967 | 0.9995 |
| RSS | 11.12726 | 4.26173 | 8.04798 | 9.3271 |
| Slope | 1.00605 | 1.00065 | 1.00324 | 1.00552 |

**Table S5.** Linear regression results for supplementary Fig. 1

**Table S6.** Linear regression results for supplementary Fig. 2

|  | **CAT + Poisson**  **3.5 ± 0.05 (Ga)** | **CAT + Poisson**  **3.8 ± 0.05** | **LG**  **3.5 ± 0.05** | **LG**  **3.8 ± 0.05** |
| --- | --- | --- | --- | --- |
| Adj. R^2^ | 0.99148 | 0.99459 | 0.99899 | 0.99912 |
| RSS | 181.73215 | 89.90161 | 32.42079 | 19.32296 |
| Slope | 1.10739 | 1.07023 | 1.05016 | 1.03444 |

**Table S7.** Linear regression results for supplementary Fig. 3

|  | **Calibration 1**  **3.5 ± 0.05 (Ga)** | **Calibration 1**  **3.8 ± 0.05** | **Calibration 2**  **3.5 ± 0.05** | **Calibration 2**  **3.8 ± 0.05** |
| --- | --- | --- | --- | --- |
| Adj. R^2^ | 0.93104 | 0.92666 | 0.96364 | 0.96462 |
| RSS | 542.67691 | 586.12708 | 488.41311 | 416.77691 |
| Slope | 0.95712 | 0.94375 | 0.97379 | 0.97422 |

**
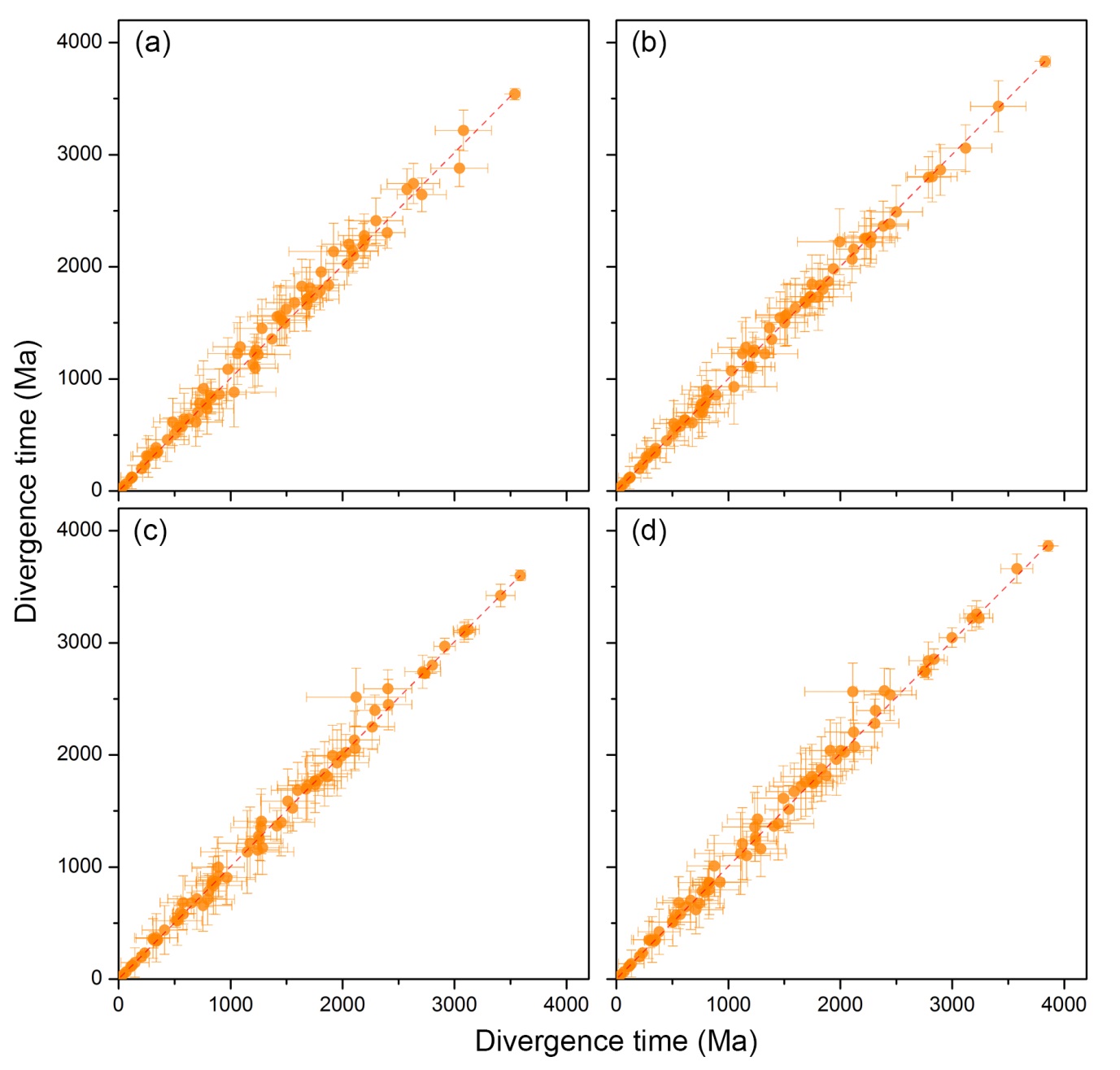
**

**Figure S1.** Effect of equilibrium frequencies model on the estimated divergence time. The estimated divergence times in the *y* axis were calculated with the GTR model, while those in the *x* axis with a uniform Poisson model. (*a*) Calculated with a root prior of 3.5 ± 0.05 Ga and using Calibration 1. (*b*) Calculated with a root prior of 3.8 ± 0.05 Ga and using Calibration 1. (*c*) Calculated with a root prior of 3.5 ± 0.05 Ga and using Calibration 2. (*d*) Calculated with a root prior of 3.8 ± 0.05 Ga and using Calibration 2

**
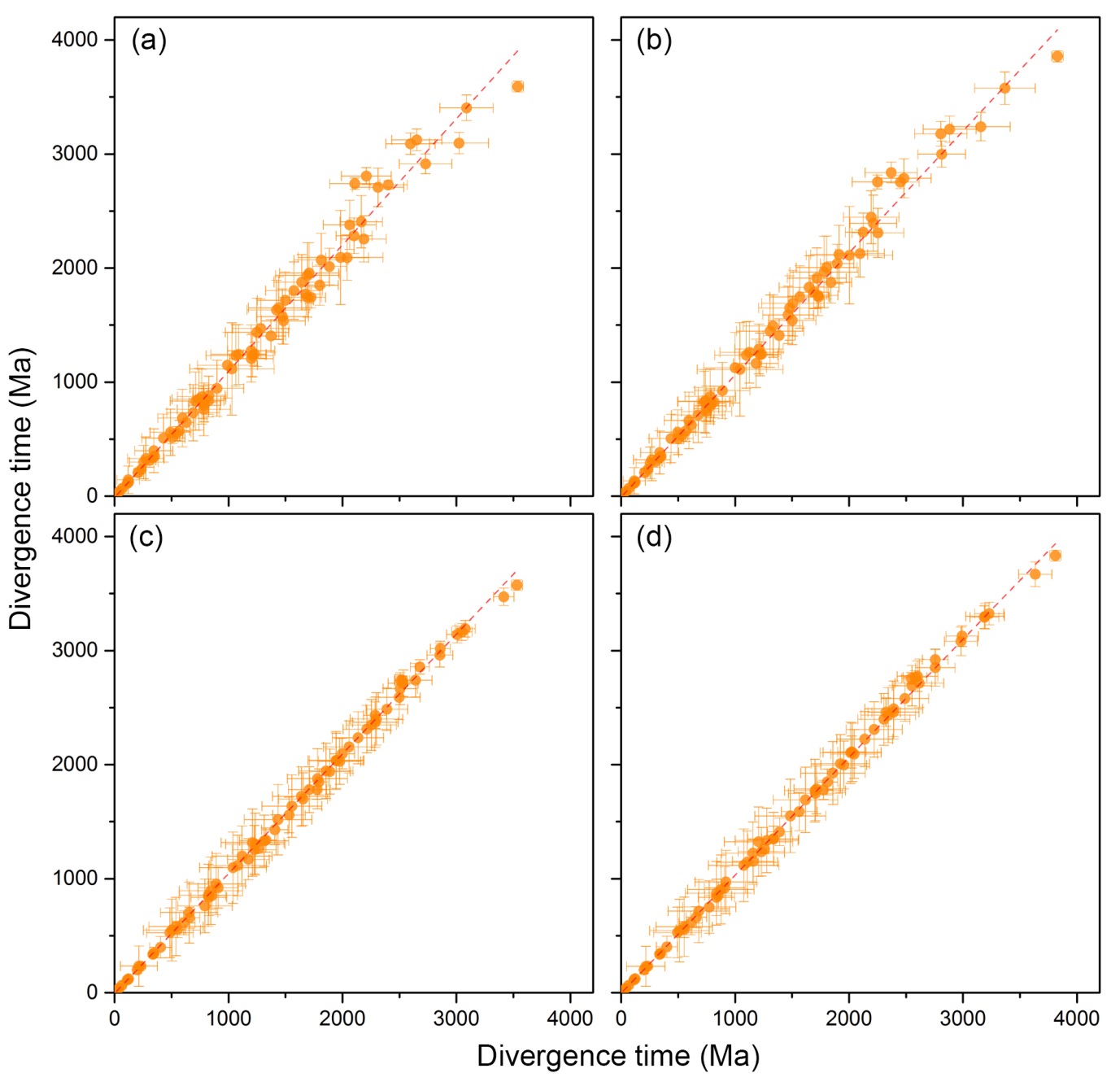
**

**Figure S2.** Effect of Calibration on the estimated divergence time. The estimated divergence times in the *y* axis were calculated using Calibration 1, while those in the *x* axis using Calibration 2. (*a*) Calculated with a root prior of 3.5 ± 0.05 Ga and under the flexible CAT + Poisson model. (*b*) Calculated with a root prior of 3.8 ± 0.05 Ga under the CAT + Poisson model. (*c*) Calculated with a root prior of 3.5 ± 0.05 Ga under the less flexible LG model. (*d*) Calculated with a root prior of 3.8 ± 0.05 Ga under the LG model

**
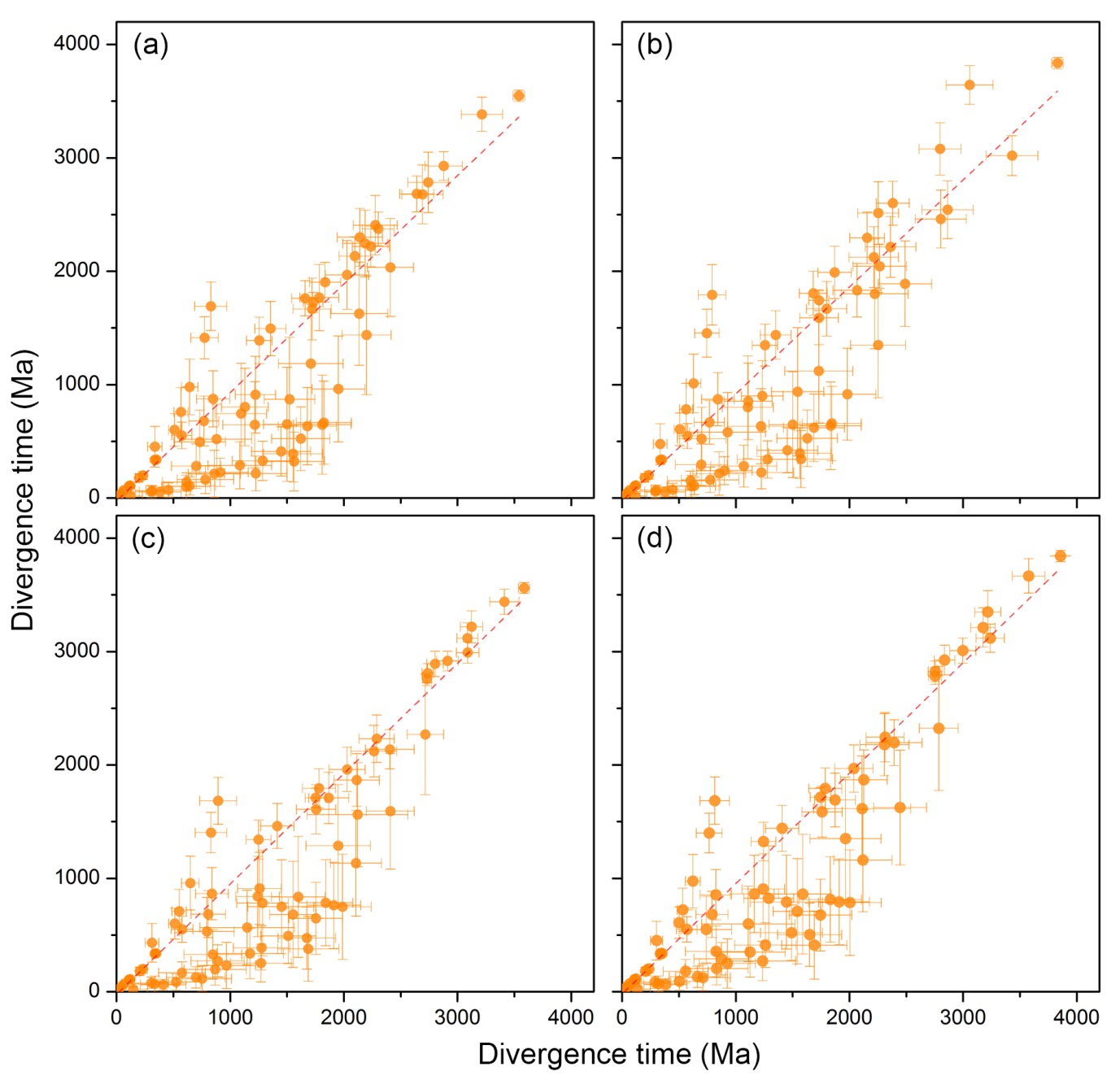
**

**Figure S3.** Effect of the model of evolutionary rate variation. The estimated divergence times in the *y* axis were calculated using an uncorrelated gamma model (CAT + Poisson), while those in the *x* axis an autocorrelated log normal model (CAT + Poisson). (*a*) Calculated with a root prior of 3.5 ± 0.05 Ga and using Calibration 1. (*b*) Calculated with a root prior of 3.8 ± 0.05 Ga and using Calibration 1. (*c*) Calculated with a root prior of 3.5 ± 0.05 Ga and using Calibration 2. (*d*) Calculated with a root prior of 3.8 ± 0.05 Ga and using Calibration 2

**References**

Bindeman, I. N., Zakharov, D. O., Palandri, J., Greber, N. D., Dauphas, N., Retallack, G. J., . . . Bekker, A. (2018). Rapid emergence of subaerial landmasses and onset of a modern hydrologic cycle 2.5 billion years ago. *Nature, 557*(7706), 545-548. doi:10.1038/s41586-018-0131-1

Boyd, E. S., Anbar, A. D., Miller, S., Hamilton, T. L., Lavin, M., & Peters, J. W. (2011). A late methanogen origin for molybdenum-dependent nitrogenase. *Geobiology, 9*(3), 221-232. doi:10.1111/j.1472-4669.2011.00278.x

Brochier-Armanet, C., Talla, E., & Gribaldo, S. (2009). The multiple evolutionary histories of dioxygen reductases: Implications for the origin and evolution of aerobic respiration. *Mol Biol Evol, 26*(2), 285-297. doi:10.1093/molbev/msn246

Brocks, J. J., Love, G. D., Summons, R. E., Knoll, A. H., Logan, G. A., & Bowden, S. A. (2005). Biomarker evidence for green and purple sulphur bacteria in a stratified Palaeoproterozoic sea. *Nature, 437*(7060), 866-870. doi:10.1038/Nature04068

Cardona, T. (2015). A fresh look at the evolution and diversification of photochemical reaction centers. *Photosynth Res, 126*(1), 111-134. doi:10.1007/s11120-014-0065-x

Cardona, T. (2018). Early Archean origin of heterodimeric Photosystem I. *Heliyon, 4*(3), e00548. doi:10.1016/j.heliyon.2018.e00548

Cardona, T., Murray, J. W., & Rutherford, A. W. (2015). Origin and evolution of water oxidation before the last common ancestor of the Cyanobacteria. *Molecular Biology and Evolution, 32*(5), 1310-1328. doi:DOI 10.1093/molbev/msv024

Clarke, J. T., Warnock, R. C. M., & Donoghue, P. C. J. (2011). Establishing a time-scale for plant evolution. *New Phytologist, 192*(1), 266-301. doi:10.1111/j.1469-8137.2011.03794.x

Cockell, C. S. (2000). Ultraviolet radiation and the photobiology of earth's early oceans. *‎Orig Life Evol Biospheres, 30*(5), 467-499.

Cornejo-Castillo, F. M., Cabello, A. M., Salazar, G., Sanchez-Baracaldo, P., Lima-Mendez, G., Hingamp, P., . . . Acinas, S. G. (2016). Cyanobacterial symbionts diverged in the late Cretaceous towards lineage-specific nitrogen fixation factories in single-celled phytoplankton. *Nat Commun, 7*. doi:10.1038/ncomms11071

David, L. A., & Alm, E. J. (2011). Rapid evolutionary innovation during an Archaean genetic expansion. *Nature, 469*(7328), 93-96. doi:10.1038/Nature09649

Ducluzeau, A.-L., & Nitschke, W. (2016). When did hemes enter the scene of life? On the natural history of heme cofactors and heme-containing enzymes. In W. A. Cramer & T. Kallas (Eds.), *Cytochrome Complexes: Evolution, Structures, Energy Transduction, and Signaling* (pp. 13-24). Dordrecht: Springer Netherlands.

Ducluzeau, A. L., Schoepp-Cothenet, B., van Lis, R., Baymann, F., Russell, M. J., & Nitschke, W. (2014). The evolution of respiratory O_2_/NO reductases: an out-of-the-phylogenetic-box perspective. *J R Soc Interface, 11*(98), 20140196. doi:10.1098/rsif.2014.0196

Duda, T. F., Jr., & Palumbi, S. R. (1999). Molecular genetics of ecological diversification: duplication and rapid evolution of toxin genes of the venomous gastropod Conus. *P Natl Acad Sci USA, 96*(12), 6820-6823.

Fischer, W. W., Hemp, J., & Johnson, J. E. (2016). Evolution of oxygenic photosynthesis. *Annu Rev Earth Planet Sci, 44*, 647-683. doi:10.1146/annurev-earth-060313-054810

Gold, D. A., Caron, A., Fournier, G. P., & Summons, R. E. (2017). Paleoproterozoic sterol biosynthesis and the rise of oxygen. *Nature, 543*(7645), 420-423. doi:10.1038/nature21412

Granold, M., Hajieva, P., Tosa, M. I., Irimie, F. D., & Moosmann, B. (2018). Modern diversification of the amino acid repertoire driven by oxygen. *P Natl Acad Sci USA, 115*(1), 41-46. doi:10.1073/pnas.1717100115

Groussin, M., & Gouy, M. (2011). Adaptation to environmental temperature is a major determinant of molecular evolutionary rates in Archaea. *Mol Biol Evol, 28*(9), 2661-2674. doi:10.1093/molbev/msr098

Heldt, D., Lawrence, A. D., Lindenmeyer, M., Deery, E., Heathcote, P., Rigby, S. E., & Warren, M. J. (2005). Aerobic synthesis of vitamin B-12: ring contraction and cobalt chelation. *Biochem Soc Trans, 33*, 815-819. doi:10.1042/Bst0330815

Innan, H., & Kondrashov, F. (2010). The evolution of gene duplications: classifying and distinguishing between models. *Nat Rev Genet, 11*(2), 97-108. doi:10.1038/nrg2689

Jordan, I. K., Wolf, Y. I., & Koonin, E. V. (2004). Duplicated genes evolve slower than singletons despite the initial rate increase. *BMC Evolutionary Biology, 4*. doi:Artn 22

10.1186/1471-2148-4-22

Kacar, B., Hanson-Smith, V., Adam, Z. R., & Boekelheide, N. (2017). Constraining the timing of the Great Oxidation Event within the Rubisco phylogenetic tree. *Geobiology, 15*(5), 628-640. doi:10.1111/gbi.12243

Knoops, B., Loumaye, E., & Van Der Eecken, V. (2007). Evolution of the peroxiredoxins. *Subcell Biochem, 44*, 27-40.

Lewis, C. A., Crayle, J., Zhou, S. T., Swanstrom, R., & Wolfenden, R. (2016). Cytosine deamination and the precipitous decline of spontaneous mutation during Earth's history. *P Natl Acad Sci USA, 113*(29), 8194-8199. doi:10.1073/pnas.1607580113

Lynch, M., & Conery, J. S. (2000). The evolutionary fate and consequences of duplicate genes. *Science, 290*(5494), 1151-1155. doi:10.1126/science.290.5494.1151

Magnabosco, C., Moore, K. R., Wolfe, J. M., & Fournier, G. P. (2018). Dating phototropic microbial lineages with reticulate gene histories. *Geobiology*. doi:10.1111/gbi.12273

Marin, J., Battistuzzi, F. U., Brown, A. C., & Hedges, S. B. (2017). The timetree of prokaryotes: New insights into their evolution and speciation. *Molecular Biology and Evolution, 34*, 437-446. doi:10.1093/molbev/msw245

Moore, R. B., Obornik, M., Janouskovec, J., Chrudimsky, T., Vancova, M., Green, D. H., . . . Carter, D. A. (2008). A photosynthetic alveolate closely related to apicomplexan parasites. *Nature, 451*(7181), 959-963. doi:10.1038/nature06635

Nakayama, T., Kamikawa, R., Tanifuji, G., Kashiyama, Y., Ohkouchi, N., Archibald, J. M., & Inagaki, Y. (2014). Complete genome of a nonphotosynthetic cyanobacterium in a diatom reveals recent adaptations to an intracellular lifestyle. *P Natl Acad Sci USA, 111*(31), 11407-11412. doi:10.1073/pnas.1405222111

Nisbet, E. G., Grassineau, N. V., Howe, C. J., Abell, P. I., Regelous, M., & Nisbet, R. E. R. (2007). The age of Rubisco: the evolution of oxygenic photosynthesis. *Geobiology, 5*(4), 311-335. doi:10.1111/j.1472-4669.2007.00127.x

Nitschke, W., van Lis, R., Schoepp-Cothenet, B., & Baymann, F. (2010). The "green" phylogenetic clade of Rieske/cytb complexes. *Photosynth Res, 104*(2-3), 347-355. doi:10.1007/s11120-010-9532-1

Ouzounis, C. A., Kunin, V., Darzentas, N., & Goldovsky, L. (2006). A minimal estimate for the gene content of the last universal common ancestor--exobiology from a terrestrial perspective. *Research in Microbiology, 157*(1), 57-68. doi:10.1016/j.resmic.2005.06.015

Pandelia, M. E., Lubitz, W., & Nitschke, W. (2012). Evolution and diversification of Group 1 [NiFe] hydrogenases. Is there a phylogenetic marker for O_2_-tolerance? *Biochim Biophys Acta, 1817*(9), 1565-1575. doi:10.1016/j.bbabio.2012.04.012

Ravin, N. V., Gruzdev, E. V., Beletsky, A. V., Mazur, A. M., Prokhortchouk, E. B., Filyushin, M. A., . . . Skryabin, K. G. (2016). The loss of photosynthetic pathways in the plastid and nuclear genomes of the non-photosynthetic mycoheterotrophic eudicot *Monotropa hypopitys*. *BMC Plant Biol, 16*(Suppl 3), 238. doi:10.1186/s12870-016-0929-7

Rosello, O. P. I., & Kondrashov, F. A. (2014). Long-term asymmetrical acceleration of protein evolution after gene duplication. *Genome Biol Evol, 6*(8), 1949-1955. doi:10.1093/gbe/evu159

Sanchez-Baracaldo, P. (2015). Origin of marine planktonic cyanobacteria. *Sci Rep, 5*, 17418. doi:10.1038/srep17418

Schopf, J. W., Kitajima, K., Spicuzza, M. J., Kudryavtsev, A. B., & Valley, J. W. (2018). SIMS analyses of the oldest known assemblage of microfossils document their taxon-correlated carbon isotope compositions. *P Natl Acad Sci USA, 115*(1), 53-58. doi:10.1073/pnas.1718063115

Shih, P. M., Hemp, J., Ward, L. M., Matzke, N. J., & Fischer, W. W. (2017). Crown group Oxyphotobacteria postdate the rise of oxygen. *Geobiology, 15*(1), 19-29. doi:10.1111/gbi.12200

Shih, P. M., Ward, L. M., & Fischer, W. W. (2017). Evolution of the 3-hydroxypropionate bicycle and recent transfer of anoxygenic photosynthesis into the Chloroflexi. *P Natl Acad Sci USA, 114*(40), 10749-10754. doi:10.1073/pnas.1710798114

Slesak, I., Slesak, H., & Kruk, J. (2012). Oxygen and Hydrogen Peroxide in the Early Evolution of Life on Earth: In silico Comparative Analysis of Biochemical Pathways. *Astrobiology, 12*(8), 775-784. doi:10.1089/ast.2011.0704

Smit, M. A., & Mezger, K. (2017). Earth's early O_2_ cycle suppressed by primitive continents. *Nature Geoscience, 10*(10), 788-792. doi:10.1038/Ngeo3030

Soo, R. M., Hemp, J., Parks, D. H., Fischer, W. W., & Hugenholtz, P. (2017). On the origins of oxygenic photosynthesis and aerobic respiration in Cyanobacteria. *Science, 355*(6332), 1436-1440. doi:10.1126/science.aal3794

Soo, R. M., Skennerton, C. T., Sekiguchi, Y., Imelfort, M., Paech, S. J., Dennis, P. G., . . . Hugenholtz, P. (2014). An expanded genomic representation of the phylum Cyanobacteria. *Genome Biol Evol, 6*(5), 1031-1045. doi:10.1093/Gbe/Evu073

Sousa, F. L., Shavit-Grievink, L., Allen, J. F., & Martin, W. F. (2013). Chlorophyll biosynthesis gene evolution indicates photosystem gene duplication, not photosystem merger, at the origin of oxygenic photosynthesis. *Genome Biol Evol, 5*(1), 200-216. doi:10.1093/Gbe/Evs127

Stolper, D. A., Revsbech, N. P., & Canfield, D. E. (2010). Aerobic growth at nanomolar oxygen concentrations. *Proc Natl Acad Sci U S A, 107*(44), 18755-18760. doi:10.1073/pnas.1013435107

Thompson, A. W., Foster, R. A., Krupke, A., Carter, B. J., Musat, N., Vaulot, D., . . . Zehr, J. P. (2012). Unicellular cyanobacterium symbiotic with a single-celled eukaryotic alga. *Science, 337*(6101), 1546-1550. doi:10.1126/science.1222700

Weiss, M. C., Sousa, F. L., Mrnjavac, N., Neukirchen, S., Roettger, M., Nelson-Sathi, S., & Martin, W. F. (2016). The physiology and habitat of the last universal common ancestor. *Nat Microbiol, 1*(9), 16116. doi:10.1038/nmicrobiol.2016.116

Yang, E. C., Boo, S. M., Bhattacharya, D., Saunders, G. W., Knoll, A. H., Fredericq, S., . . . Yoon, H. S. (2016). Divergence time estimates and the evolution of major lineages in the florideophyte red algae. *Sci Rep, 6*, 21361. doi:10.1038/srep21361

Zamocky, M., Janecek, S., & Koller, F. (2000). Common phylogeny of catalase-peroxidases and ascorbate peroxidases. *Gene, 256*(1-2), 169-182. doi:10.1016/S0378-1119(00)00358-9
